# BMAL1 Dysregulation as a Contributing Mechanism Linking Obesity to Oocyte and Endometrial Dysfunction in IVF

**DOI:** 10.3390/ijms27188001

**Published:** 2026-09-08

**Authors:** Charalampos Voros, Fotios Chatzinikolaou, George Papadimas, Ioannis Papapanagiotou, Nektaria Zagorianakou, Ali Can Gunes, Athanasios Karpouzos, Kyriakos Bananis, Charalampos Tsimpoukelis, Maria Anastasia Daskalaki, Stylianos Makrydimas, Ioannis Pikrides, Nikolaos Thomakos, Panagiotis Antsaklis, Dimitrios Loutradis, Georgios Daskalakis

**Affiliations:** 1 Department of Obstetrics and Gynecology, ‘Alexandra’ General Hospital, National and Kapodistrian University of Athens, 80 Vasilissis Sofias Avenue, 11528 Athens, Greecepikrides114@gmail.com (I.P.);; 2 Laboratory of Forensic Medicine and Toxicology, School of Medicine, Aristotle University of Thessaloniki, 54124 Athens, Greece; 3 Athens Medical School, National and Kapodistrian University of Athens, 15772 Athens, Greeceloutradi@otenet.gr (D.L.); 4 Scientific Laboratory for Innovative Technologies in Internal Medicine, Preventive Medicine and Overall Care, Department of Nursing, School of Health Sciences, University of Ioannina, 45500 Ioannina, Greece; 5 Department of Obstetrics and Gynecology, Faculty of Medicine, Hacettepe University, Ankara 06100, Turkey; 6 King’s College Hospitals NHS Foundation Trust, London SE5 9RS, UK; 7 Medical School, Aristotle University of Thessaloniki, 54124 Thessaloniki, Greece

**Keywords:** circadian clock, BMAL1, obesity, granulosa cells, endometrial receptivity, oocyte quality, melatonin, window of implantation

## Abstract

Obesity affects nearly one in three women of reproductive age worldwide and consistently reduces success rates in in vitro fertilization, yet the molecular basis for this reduction remains fragmented across separate lines of evidence. Circadian clock genes, particularly BMAL1, orchestrate metabolic and reproductive physiology through transcription–translation feedback loops present in adipose tissue, ovarian granulosa cells, and endometrial stroma. Adiposity-driven metabolic shifts, including altered PPAR-γ signaling and reduced glutamine–methionine uptake, degrade BMAL1 expression and flatten its rhythmic oscillation in peripheral tissues. Within granulosa cells, loss of BMAL1 rhythmicity impairs mitochondrial biogenesis and disrupts UPRmt-mediated proteostasis, driving reactive oxygen species accumulation and compromising oocyte competence. Parallel disruption of clock-controlled transcription factors in endometrial epithelium and stroma is proposed to alter decidualization programs and displace the window of implantation, which would produce a receptivity defect independent of oocyte quality if confirmed directly in human tissue. Clinical data are consistent with a dual mechanism: obese women undergoing donor-oocyte cycles—where oocyte quality is controlled for—show reduced implantation rates in several but not all cohorts—a pattern compatible with an endometrial contribution distinct from oocyte-level damage, rather than proof of it. Synthesizing evidence from adipocyte biology, ovarian physiology, and endometrial receptivity research drawn largely from rodent models, cultured cell systems, and observational human cohorts, this review proposes BMAL1 dysregulation as a candidate unifying mechanism connecting obesity to impaired IVF outcomes at the gametic and uterine level, while acknowledging that direct causal evidence in humans is still lacking. Chronotherapeutic strategies, including melatonin supplementation and the timing of weight-loss interventions relative to ovarian stimulation, are discussed as hypotheses for future testing rather than current clinical recommendations. BMAL1 dysregulation is presented here as one candidate contributor among several interacting mechanisms, and any translational strategy would need to be part of a broader, coordinated approach to obesity-related IVF failure rather than a stand-alone intervention.

## 1. Introduction

Obesity now affects close to a third of women of reproductive age in industrialized countries, and its consequences for fertility extend well beyond ovulatory dysfunction. Live birth rates after in vitro fertilization fall by roughly 5–7% for every five-unit rise in body mass index, and miscarriage risk climbs in parallel, patterns documented across multiple large cohorts and consistently reproduced regardless of stimulation protocol [1].

Mechanistic explanations have tended to converge on two competing hypotheses: a primary oocyte defect driven by lipotoxicity and mitochondrial compromise, or a primary endometrial defect rooted in altered steroid signaling and chronic low-grade inflammation [2]. Neither hypothesis alone accounts for the full clinical picture, and studies using the donor-oocyte model where oocyte quality is held constant by using young, healthy donors have produced genuinely conflicting results, with some cohorts showing no effect of recipient BMI on implantation and others, including the largest single-center series published to date, showing a clear and dose-dependent decline [3,4].

A parallel and largely separate body of work has established that circadian biology operates well beyond the suprachiasmatic nucleus. Peripheral molecular clocks, built around the transcription–translation feedback loop of BMAL1, CLOCK, PER, and CRY, run autonomously in adipose tissue, granulosa cells, and endometrial stroma, each tuned to local physiological demands while remaining sensitive to systemic metabolic cues [5]. Disruption of this peripheral clock network in obesity is well characterized at the level of white adipose tissue: reduced PPAR-γ activity in obese adipocytes suppresses the glutamine/methionine transporter SLC1A5, lowering the epigenetic substrates required for histone acetylation and methylation at the Bmal1 promoter and flattening its rhythmic expression [6]. What has received far less attention is whether this same chronodisruption propagates into the reproductive tissues most relevant to IVF outcome, and whether it might explain the very inconsistency that has frustrated the oocyte-versus-endometrium debate for two decades.

Isolated observations point toward exactly this possibility. BMAL1 is required for normal steroidogenic gene expression in human luteinized granulosa cells, correlating directly with CYP11A1, CYP19A1, STAR, and ESR2 transcription [7], while its loss in decidualizing endometrial stromal cells impairs trophoblast invasion through dysregulated TIMP3 expression and has been linked independently to recurrent miscarriage [8]. Melatonin, itself a downstream output and upstream modulator of the same clock machinery, improves oocyte and embryo quality in several clinical trials, offering indirect but consistent support for a functional circadian axis operating within the ovary and uterus during assisted reproduction [9,10]. No existing review, however, has assembled these threads into a single mechanistic account linking obesity-driven BMAL1 suppression in adipose tissue to its downstream consequences in the oocyte and endometrium as parts of one continuous pathway.

A caveat is needed here. Much of this evidence comes from rodent models and cultured cells, where BMAL1 can be manipulated directly. No study has yet measured this same two-mechanism model directly in ovarian tissue from obese women. The picture holds together across cell lines and mice. It has not yet been shown in the tissue that matters most for this review’s argument. Causality in these systems is well supported. The human data are mostly observational, and the donor-oocyte literature remains genuinely mixed. This review treats BMAL1 dysregulation as a testable hypothesis, not an established mechanism in human reproduction. We flag throughout which claims rest on direct experimental evidence and which rest on association alone.

**Figure 1:**
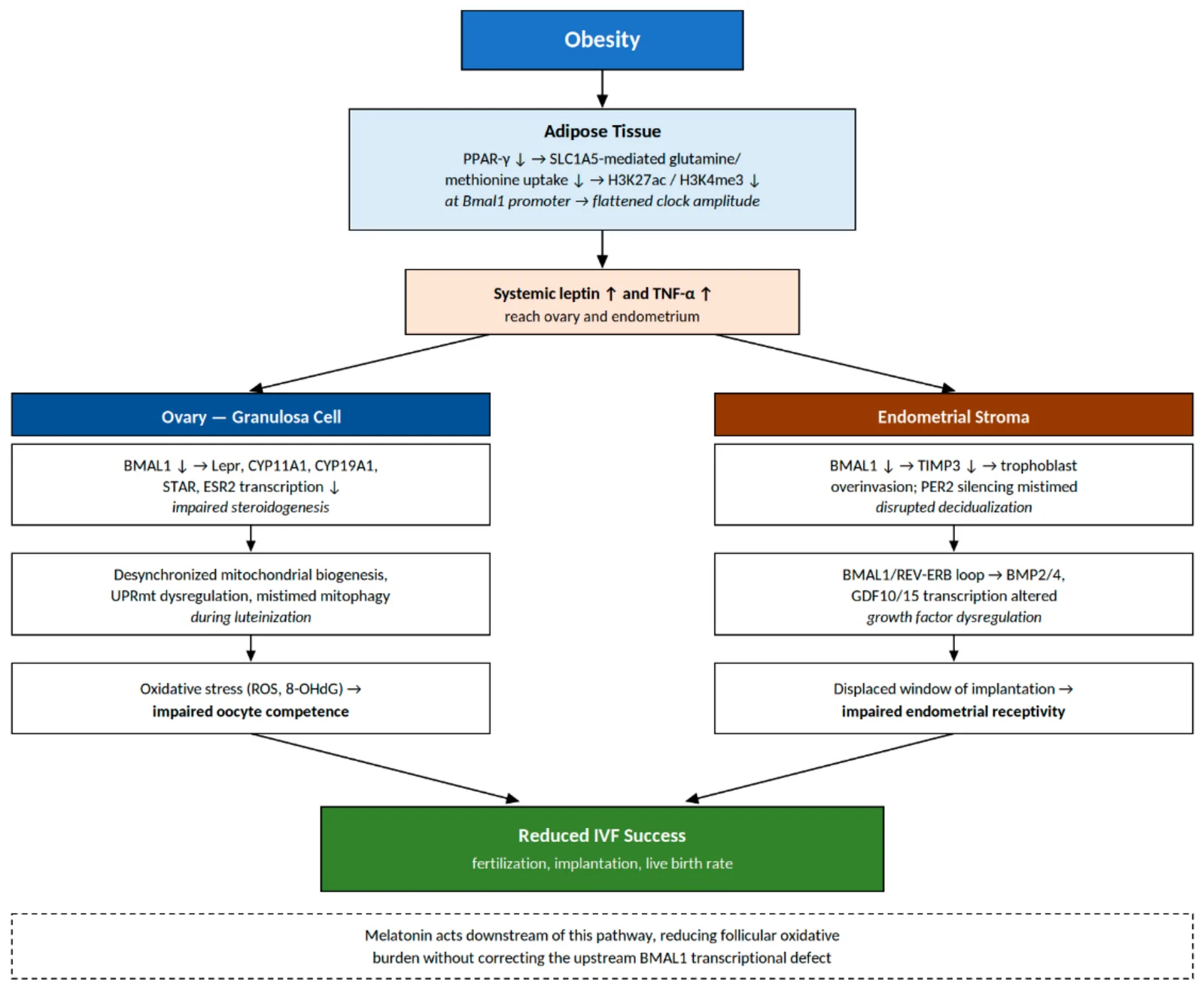
Obesity-Driven BMAL1 Disruption in IVF. Schematic overview of the proposed mechanism linking obesity-driven circadian disruption to impaired IVF outcome. Adipocyte PPAR-γ suppression flattens Bmal1 promoter activity, and the resulting rise in circulating leptin and TNF-α propagates the disruption to granulosa cells and endometrial stroma through distinct but convergent molecular routes, both feeding into reduced fertilization, implantation, and live-birth rates. The dotted box represents melatonin’s proposed point of intervention in this pathway. Melatonin acts both as a chronobiotic signal capable of resetting peripheral clock phase and as a direct antioxidant within the follicular compartment, independent of any restoration of Bmal1 transcription itself. It is shown as a separate, dotted module because its mechanism runs parallel to, rather than through, the core PPAR-γ/SLC1A5/Bmal1 pathway traced by the solid arrows, consistent with the distinction discussed in Section 7.1.

Addressing that gap is the purpose of the present review. Adipocyte clock disruption in obesity is traced through its molecular consequences in granulosa cell steroidogenesis and mitochondrial function, then through its parallel effects on endometrial decidualization and receptivity, before returning to the clinical literature to ask whether this shared mechanism can account for the divergent findings reported in the donor-oocyte model [11]. Melatonin supplementation is considered separately as a chronobiotic intervention with direct translational relevance, given its position at the intersection of the pathways described throughout the review. Figure 1 provides a schematic overview of this proposed mechanism, tracing the pathway from adipocyte clock disruption through its parallel consequences in the ovary and endometrium to the shared clinical endpoint discussed in the remainder of this review.

## 2. Materials and Methods

Literature searches were performed across PubMed/MEDLINE, Scopus, Web of Science, and the Cochrane Library for the period January 2000 through July 2026, a start date chosen because it follows the cloning of mammalian Bmal1 and the establishment of peripheral clock biology as a field distinct from central pacemaker research. Search terms fell into three groups that were combined through Boolean operators: circadian biology terms (“*BMAL1*”, “ARNTL”, “circadian clock”, “CLOCK gene”, “PER1”, “PER2”, “CRY1”, “CRY2”, “REV-ERB”, “NR1D1”, “peripheral clock”, “chronodisruption”); metabolic terms (“obesity”, “adipose tissue”, “adipocyte”, “PPAR-gamma”, “leptin”, “insulin resistance”); and reproductive terms (“granulosa cell”, “oocyte quality”, “oocyte competence”, “mitochondrial dysfunction”, “endometrial receptivity”, “decidualization”, “window of implantation”, “trophoblast invasion”, “in vitro fertilization”, “donor oocyte”, “melatonin”). Representative combinations included (“BMAL1” OR “circadian clock”) AND (“obesity” OR “adipocyte”) AND (“oocyte” OR “granulosa cell”), and (“circadian clock” OR “BMAL1”) AND (“endometrial receptivity” OR “decidualization” OR “implantation”). Searches were repeated at intervals throughout manuscript preparation, with the final update carried out in July 2026.

Eligible articles included original research, narrative reviews, systematic reviews, and meta-analyses published in English in peer-reviewed journals. Human and murine studies were both accepted where they examined clock gene function at the molecular level in adipose tissue, granulosa or theca cells, or endometrial stroma and epithelium. Clinical studies qualified if they reported IVF, oocyte-donation, or embryo-transfer outcomes stratified by body mass index, or if they evaluated melatonin supplementation in assisted reproduction through randomized, non-randomized, or cohort designs. Conference abstracts lacking a subsequent full publication, non-peer-reviewed preprints, case reports with fewer than five subjects, and non-English articles without an available translation were excluded. Male-factor circadian studies, metabolic or cardiovascular circadian pathology without a reproductive endpoint, and shift-work epidemiology lacking any molecular component were excluded from the mechanistic sections, though a limited number of shift-work epidemiological studies were kept in the Introduction to support the broader link between environmental circadian disruption and reproductive dysfunction.

Reference lists of all retrieved articles were hand-searched for additional primary sources a needed step, as indexing terms differ considerably across the adipose biology, reproductive endocrinology, and chronobiology literature, and a purely electronic search risks missing relevant work published under unfamiliar terminology in any one of these fields. Given the mechanistic and cross-disciplinary scope of the topic, spanning adipocyte physiology, ovarian biology, and endometrial receptivity, a systematic review methodology with formal risk-of-bias scoring was not attempted. What follows is a narrative synthesis built to integrate mechanistic and clinical findings from these three domains into a single working model, and the absence of a pre-registered protocol or formal quality scoring is acknowledged as a limitation in the Discussion.

## 3. Molecular Disruption of BMAL1 in Obese Adipose and Peripheral Tissue

### 3.1. The Peripheral Circadian Clock in White Adipose Tissue

Adipose tissue was long treated as a passive energy reservoir, a view abandoned once white adipose tissue was shown to harbor a fully functional peripheral circadian clock built on the same transcription–translation feedback loop found in the suprachiasmatic nucleus [12]. BMAL1 heterodimerizes with CLOCK to drive E-box-dependent transcription of Per1, Per2, Cry1, and Cry2, whose protein products accumulate through the day, translocate back into the nucleus, and inhibit the very BMAL1:CLOCK complex responsible for their own synthesis. This negative feedback arm generates an autoregulatory cycle with a period close to twenty-four hours, and it does not operate alone [13]. A second, interlocking loop runs through Rev-erbα and the ROR family of nuclear receptors, both of which compete for binding at RORE elements within the Bmal1 promoter, Rev-erbα repressing transcription and the RORs activating it. The two loops together buffer the system against noise and give the clock its characteristic robustness, a property that becomes relevant later when considering how much metabolic insult is required to meaningfully degrade the oscillation rather than merely shift its phase [14].

In white adipose tissue, this machinery is not a vestigial echo of central clock function but an active regulator of local physiology. Rhythmic Bmal1 expression governs downstream genes controlling lipogenesis, lipolysis, and the timed secretion of adipokines, meaning the adipocyte’s metabolic behavior itself follows a daily program rather than remaining constant. Triglyceride synthesis and release, free fatty acid efflux into the circulation, and the transcription of leptin and adiponectin all show time-of-day dependence that traces back to this local oscillator rather than to systemic signals alone. The amplitude of Bmal1 oscillation measured in adipose tissue is comparable to that recorded in the liver, one of the best-characterized peripheral clock tissues, underscoring that adipose tissue is not a minor or attenuated oscillator within the broader circadian network but a full participant in it [11].

Obesity disrupts this rhythm at the level of amplitude rather than period, a distinction that matters mechanistically. A shortened or lengthened period would suggest a defect in the core feedback kinetics of the loop itself, altering PER or CRY degradation rates, for instance, whereas a flattened amplitude with preserved period points instead toward a problem in the transcriptional drive feeding into an otherwise intact oscillator [15]. Comparisons of ob/ob mice, diet-induced obese mice, and obese human subjects consistently show this pattern: a marked reduction in BMAL1 expression within white adipose tissue relative to lean controls documented across a full six-point circadian time series spanning CT2 through CT22 with the flattening most pronounced at what would normally be the circadian peak of expression rather than a uniform depression spread evenly across the cycle [16]. The same attenuation extends to Rev-erbα, Rev-erbβ, Per1, and Cry1, indicating that obesity degrades the entire feedback architecture rather than selectively silencing a single node within it, which argues against a narrow, single-gene explanation and toward a systemic failure of the transcriptional inputs sustaining the loop as a whole.

This distinction between amplitude loss and period disruption also has a practical implication for how the mechanism should be studied and eventually targeted therapeutically. Interventions aimed at restoring period fidelity, such as chronotype-matched light exposure or melatonin timing, would be expected to have limited effect if the underlying defect is upstream transcriptional drive rather than the oscillator’s internal kinetics [17]. Restoring the amplitude of BMAL1 expression instead requires addressing whatever is suppressing its transcriptional inputs in the first place, which is the question taken up directly in the following subsection through the PPAR-γ/SLC1A5 axis. The adipose clock, in other words, is not broken in the sense of running at the wrong speed. Ιt is broken in the sense of running too quietly, its peaks blunted and its capacity to entrain downstream metabolic and reproductive tissue correspondingly diminished [16].

Whether this blunted rhythm is a cause or a consequence of obesity remains a genuinely open question, and the honest answer is probably both, operating in a loop rather than a single direction. Genetic disruption of Bmal1 in otherwise lean animals produces an obese phenotype, establishing that clock loss alone is sufficient to drive weight gain and metabolic dysfunction independent of any prior obesogenic diet [15]. At the same time, diet-induced obesity produces the clock disruption described above even in animals with an intact genetic clock, establishing the converse direction just as clearly. The most parsimonious interpretation is a bidirectional relationship in which an initial insult, whether genetic, dietary, or behavioral, degrades adipocyte clock amplitude, and the resulting metabolic dysfunction further erodes the transcriptional inputs sustaining that same clock, entrenching a self-perpetuating cycle rather than a linear cause-and-effect chain [18]. This framing matters for the reproductive consequences developed later in this review, because it implies that BMAL1 disruption in adipose tissue is unlikely to resolve quickly or fully with weight loss alone, a point returned to in the Discussion when considering the timing of bariatric intervention relative to ovarian stimulation.

### 3.2. The PPAR-γ/SLC1A5 Axis and Epigenetic Silencing of Bmal1

Mechanistically, the amplitude defect described above traces back to PPAR-γ acting as an upstream integrator between metabolic state and clock gene transcription, and the pathway connecting the two has been worked out in considerable molecular detail. PPAR-γ is a nuclear receptor best known for its role in adipocyte differentiation and insulin sensitization, and its activity in white adipose tissue is measurably reduced in the obese state, driven in part by chronic inflammatory signaling discussed in the following subsection [16]. What links PPAR-γ to the circadian machinery is its direct transcriptional control over SLC1A5, a transporter for which PPAR-γ binds a response element within the promoter and drives its expression under normal metabolic conditions. Falling PPAR-γ activity in obesity therefore produces a direct and measurable downregulation of SLC1A5 expression in adipose tissue, an effect confirmed through a systematic screen of transport and metabolic genes altered in obese white adipose tissue, from which SLC1A5 emerged as the principal transporter responsible for the downstream metabolic consequences described below [16].

SLC1A5 functions as a dual transporter mediating cellular uptake of glutamine and methionine; at first glance, these two amino acids appear unrelated to circadian transcription. Their relevance becomes clear once their downstream metabolic fates are considered [19]. Glutamine entering the adipocyte is metabolized through the tricarboxylic acid cycle into acetyl-CoA, the essential two-carbon donor required by histone acetyltransferases for the addition of acetyl groups to lysine residues on histone tails. Methionine, independently, is converted into S-adenosylmethionine through the methionine cycle, and S-adenosylmethionine serves as the universal methyl donor employed by histone methyltransferases for the addition of methyl groups to those same histone tails [20]. Reduced SLC1A5-mediated uptake of both amino acids in obese adipose tissue therefore constrains the cell’s capacity to generate both classes of epigenetic substrate simultaneously, denying the transcriptional machinery the acetyl-CoA and SAM pools it would otherwise draw on to open chromatin at actively transcribed loci.

The Bmal1 promoter appears to be particularly sensitive to this substrate restriction. Reduced glutamine and methionine availability in obese white adipose tissue is associated with measurably decreased H3K27ac and H3K4me3 marks specifically at the Bmal1 promoter region, two modifications strongly associated with active, permissive chromatin and transcriptional accessibility [21]. Both marks depend directly on the acetyl-CoA and SAM pools whose generation has just been described as compromised, giving a direct biochemical explanation for why Bmal1 transcription specifically, rather than housekeeping gene expression broadly, shows such sensitivity to this metabolic constraint. Promoters with lower baseline reliance on these particular histone marks, or with more redundant activating inputs, would be expected to tolerate the same substrate restriction with less consequence, which may partly explain why the clock feedback loop is disproportionately affected relative to other adipocyte transcriptional programs under the same metabolic conditions [22].

Within this adipocyte system, the causal architecture of the pathway has been demonstrated experimentally rather than merely inferred from correlation, an important distinction given how much the circadian–metabolic literature rests on associative rather than interventional evidence, though this experimental confirmation comes from cultured adipocytes and mouse models rather than from human adipose tissue in vivo. Exogenous glutamine or methionine administration, delivered either to cultured adipocytes in vitro or systemically to obese mice in vivo, restores both H3K27ac and H3K4me3 marks at the Bmal1 promoter toward levels seen in lean tissue, and this epigenetic restoration is accompanied by corresponding rescue of Bmal1 transcription itself [16]. The experiment closes the causal loop from PPAR-γ through SLC1A5, amino acid availability, and histone modification to Bmal1 transcription. This is conducted bidirectionally: restoring the substrate restores the mark, and restoring the mark restores the transcript.

What emerges from this sequence is not a simple linear cascade but a self-reinforcing loop with genuine explanatory power for the clinical persistence of obesity-related metabolic dysfunction. Obesity suppresses PPAR-γ activity. PPAR-γ suppression degrades SLC1A5-dependent glutamine and methionine uptake; reduced uptake starves the epigenetic machinery maintaining Bmal1 promoter accessibility, and the resulting clock disruption has itself been shown experimentally to promote further adipogenic differentiation and fat accumulation, closing the cycle back onto its own starting point [23]. This closed-loop architecture offers one plausible molecular explanation for why obesity, once metabolically established, tends toward self-perpetuation rather than easy reversal through caloric correction alone, since the epigenetic state degrading Bmal1 transcription itself is a consequence of the very adiposity it helps sustain. The therapeutic implication, addressed later in this review in the context of bariatric surgery timing relative to IVF stimulation, is that weight loss achieved through caloric restriction may not immediately restore the amino acid flux and epigenetic marks described here, since adipocyte-intrinsic epigenetic states can persist beyond the resolution of the systemic metabolic phenotype that originally produced them [24].

### 3.3. Inflammatory Convergence on the Same Transcriptional Node

Chronic low-grade inflammation compounds this mechanism rather than acting as an independent parallel pathway operating alongside it, and the distinction matters because it changes how the two insults should be conceptualized when designing interventions [25]. Obese adipose tissue is characterized by substantial macrophage infiltration, a hallmark of the obese phenotype first described at the histological level and since confirmed at the level of cytokine output, with infiltrating macrophages within hypertrophic fat depots secreting TNF-α at levels well above those found in lean adipose tissue [26]. TNF-α signaling through its receptor activates downstream kinase cascades, and one well-established consequence of this activation is phosphorylation-dependent inhibition of PPAR-γ’s DNA-binding capacity, reducing its ability to occupy response elements at target promoters, including that of SLC1A5.

**Figure 2:**
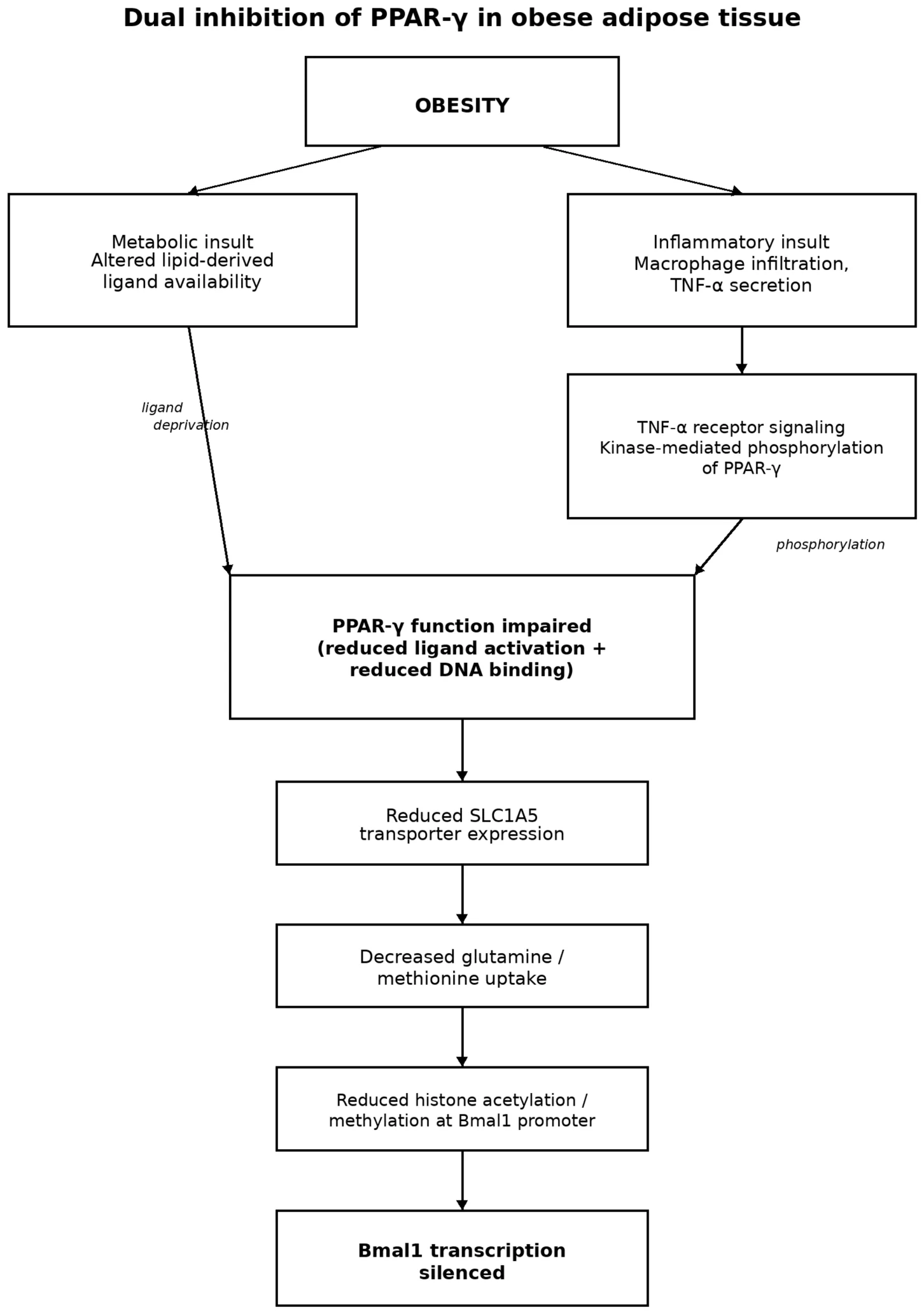
Dual inhibition of PPAR-γ in obese adipose tissue. A metabolic insult (altered lipid-derived ligand availability) and an inflammatory insult (macrophage-derived TNF-α triggering kinase-mediated phosphorylation of PPAR-γ) act through distinct biochemical routes but converge on the same target, PPAR-γ function. Impaired PPAR-γ reduces SLC1A5 transporter expression, lowering glutamine and methionine uptake, which, in turn, reduces histone acetylation and methylation marks required to keep the Bmal1 promoter active, silencing Bmal1 transcription.

The practical consequence is that obese adipose tissue faces what amounts to a double hit on PPAR-γ function rather than two separate and additive problems, summarized schematically in Figure 2. One insult is metabolic, arising directly from the obese state itself through mechanisms not yet fully characterized but likely involving lipid-derived ligand availability for the receptor [27]. The second insult is inflammatory, arising from the immune infiltrate that obesity itself recruits into the tissue, and this second insult acts through a distinct biochemical mechanism, namely kinase-mediated phosphorylation rather than ligand deprivation, yet converges on the identical downstream target. Both pathways intersect at PPAR-γ’s capacity to activate SLC1A5, meaning the amino acid restriction and subsequent epigenetic silencing of Bmal1 described in the preceding subsection receives reinforcing pressure from two mechanistically independent directions rather than a single vulnerable point that might be more easily corrected [28].

This convergence has an important implication for intervention strategy, one that recurs when the clinical and therapeutic sections of this review are reached. An approach targeting only the metabolic insult, such as dietary glutamine or methionine supplementation intended to bypass the SLC1A5 bottleneck directly, would be expected to show partial rather than complete restoration of Bmal1 transcription if the inflammatory suppression of PPAR-γ remains active and continues to constrain SLC1A5 expression through the kinase-mediated route independently of substrate availability [29]. Conversely, an anti-inflammatory approach alone, without addressing the metabolic substrate depletion, would leave the glutamine/methionine restriction in place even if PPAR-γ phosphorylation were reduced, since PPAR-γ activity restored by resolving inflammation still requires adequate substrate flux downstream to translate into meaningful histone mark restoration. The two-hit architecture therefore argues for combined strategies, whether pharmacological, nutritional, or surgical, that address both the metabolic and inflammatory arms simultaneously rather than assuming either alone will fully normalize the adipocyte clock [30].

The broader relevance of this inflammatory convergence to reproductive tissue lies in the fact that TNF-α and related pro-inflammatory cytokines are not confined to adipose tissue in the obese state but circulate systemically and reach the ovary and endometrium directly. Follicular fluid from obese women undergoing IVF has been characterized as showing an altered cytokine profile relative to lean controls, and endometrial tissue in obesity likewise shows evidence of a pro-inflammatory local environment. Whether the same PPAR-γ-mediated mechanism operates locally within granulosa cells or endometrial stroma, independent of any adipose-derived signal, or whether the reproductive tissue clock disruption described in the following two sections of this review is instead a downstream consequence of circulating inflammatory mediators originating in adipose tissue, remains an open mechanistic question that current evidence cannot fully resolve. Both possibilities are compatible with the broader argument developed in this review, since either would establish inflammation as a second, parallel route by which obesity reaches reproductive tissue clock function alongside the adipokine-mediated route described in the next subsection.

### 3.4. Adipokine-Mediated Propagation to Reproductive Tissue

Circulating adipokines extend adipocyte clock disruption beyond the boundaries of adipose tissue itself and into distant reproductive organs, offering the clearest available molecular bridge between the mechanism described above and the ovarian and endometrial consequences addressed in the sections that follow. Leptin is the most extensively characterized of these adipokines in this context. Secreted by adipocytes in direct proportion to fat mass, leptin is chronically elevated in the obese state, and its relevance to circadian biology extends well beyond its established role in hypothalamic appetite regulation. The leptin receptor gene itself, Lepr, carries E-box elements within its promoter region, meaning Lepr transcription is subject to direct circadian control by the BMAL1:CLOCK heterodimer rather than being regulated purely through leptin-independent mechanisms [31].

In ovarian granulosa cells specifically, this circadian control of Lepr has been demonstrated directly, with BMAL1 shown to regulate Lepr expression in granulosa cells and disruption of this regulation altering downstream estrogen synthesis through the leptin receptor signaling cascade [32]. The finding establishes something more specific than a general association between obesity, elevated leptin, and impaired fertility. Ιt identifies a molecular loop in which the granulosa cell’s own circadian clock governs its sensitivity to circulating leptin by controlling Lepr transcription, while the leptin level to which that receptor responds is itself set by systemic adiposity and, per the preceding subsections, by an adipocyte clock already degraded by obesity. The ovary’s response to the metabolic environment is therefore not simply a matter of leptin concentration acting on a fixed receptor population, but a matter of two interacting oscillators: the adipocyte clock setting leptin output and the granulosa cell clock setting Lepr sensitivity, both of which are compromised simultaneously in the obese state [33].

The implications of this bidirectional loop are best understood by considering what happens when both halves of the circuit are disrupted together rather than considering leptin elevation and granulosa clock disruption as separate insults. If granulosa cell BMAL1 rhythmicity were intact despite elevated systemic leptin, Lepr expression would still follow its normal circadian pattern, and the cell’s sensitivity to the elevated hormone would at least remain predictable and rhythmically timed, even if the absolute leptin exposure were abnormal [32]. Conversely, if leptin levels were normal despite granulosa cell BMAL1 disruption, Lepr transcription would lose its circadian patterning, but it would, at least, respond to a physiological hormone concentration. The obese state, as characterized by the evidence reviewed here, appears to combine both insults: elevated leptin acting on a receptor whose own circadian expression pattern is itself compromised by the same systemic clock disruption responsible for the elevated hormone level in the first place. This compounding of insults, rather than either alone, offers a more complete account of why granulosa cell steroidogenic function proves so consistently disrupted in obesity-associated infertility, a theme developed further once the granulosa cell mechanisms are addressed directly in the following section [31].

Systemic propagation of adipocyte clock disruption through this adipokine route offers a plausible and mechanistically grounded explanation for how whole-body obesity reaches tissues with no direct metabolic overlap with fat storage itself [34]. Peripheral clocks across the organism do not function as isolated, independently ticking oscillators; their mutual entrainment depends on shared circulating cues, including glucocorticoid rhythms set by hypothalamic–pituitary–adrenal axis activity, feeding-related signals tied to meal timing and nutrient absorption, core body temperature fluctuations that vary systematically across the day, and the adipokine and cytokine profile characteristic of the individual’s metabolic state as detailed in the preceding subsection [35]. A degraded rhythm in one major peripheral tissue, particularly one as metabolically active and as large in mass as adipose tissue in the obese state, does not remain contained within that tissue’s own boundaries, since it necessarily reshapes the entrainment signals reaching every downstream organ sensitive to those same circulating cues [34].

Adipose tissue, given both its mass and its secretory activity in the obese state, is therefore positioned to function as an amplifier of chronodisruption across the organism rather than merely a passive site of local clock failure confined to its own tissue boundaries. Elevated circulating leptin in particular supplies a direct, well-characterized molecular conduit by which the disrupted adipocyte clock reaches the ovary through the Lepr–BMAL1 loop described above, while the inflammatory cytokines discussed in the previous subsection offer a second, parallel conduit whose tissue-level consequences remain less fully mapped. Whether either or both of these conduits extend, with comparable directness, into the endometrium, as well as what specific molecular consequences follow once BMAL1 signaling is degraded within the oocyte-supporting granulosa cell compartment and within the endometrial stromal tissue, respectively, are the questions directly addressed in the following two sections.

## 4. Molecular Mechanisms in Granulosa Cells and Oocyte Competence

### 4.1. BMAL1 as a Direct Regulator of Steroidogenic Gene Transcription

The circadian clock’s presence in granulosa cells was established well before its functional consequences were understood, and it is worth being precise about what “presence” means at this level, since granulosa cells are not simply passive recipients of gonadotropin signaling but carry an autonomous oscillator capable of local transcriptional control independent of pituitary input [36]. Human luteinized granulosa cells, when synchronized in culture using dexamethasone, show clear rhythmic oscillation of the core clock genes, with BMAL1, CLOCK, PER1, PER2, CRY1, and CRY2 all cycling with the expected antiphase relationship between the positive and negative arms of the feedback loop. This establishes that granulosa cells are not merely exposed to circadian signals arriving from elsewhere but generate and maintain their own rhythm at the cellular level, a distinction that matters because it means the disruption described in the preceding section, arriving through leptin and inflammatory mediators from disrupted adipose tissue, is landing on a tissue that already has its own vulnerable oscillator rather than one that is circadian-naive and therefore passively shielded from the consequences of dysregulation elsewhere [37].

The functional significance of this local clock became clear once BMAL1 expression was manipulated directly rather than merely observed. Knockdown of BMAL1 using siRNA in KGN cells, an established human granulosa-like tumor cell line frequently used as a model for luteinized granulosa cell physiology, produces a significant decrease in the expression of the core steroidogenic enzymes CYP11A1, CYP19A1, STAR, and ESR2 [7]. CYP11A1 catalyzes the rate-limiting cholesterol side-chain cleavage step initiating all downstream steroid hormone synthesis; STAR mediates cholesterol transport into the mitochondrial inner membrane where that cleavage occurs. CYP19A1 encodes aromatase, responsible for the final conversion of androgens to estrogens, and ESR2 encodes the estrogen receptor beta isoform through which much of the resulting estradiol signal is transduced locally within the follicle [38]. Loss of BMAL1 therefore does not selectively impair one point along the steroidogenic pathway but depresses expression at multiple sequential steps simultaneously, from cholesterol delivery through final estrogen receptor signaling, indicating that BMAL1 functions as something closer to a master enabler of the entire steroidogenic program rather than a regulator of any single enzymatic step within it [39].

The reciprocal experiment confirms the direction of this relationship rather than leaving it as a correlative inference from knockdown alone. BMAL1 overexpression in both KGN cells and HGL5 cells, a second human granulosa-lutein cell line used to cross-validate findings against potential line-specific artifacts, produces a corresponding increase in CYP11A1 and CYP19A1 expression, with the overexpression experiments further demonstrating that BMAL1 positively controls 17β-estradiol secretion at the level of measurable hormone output rather than transcript abundance alone [7]. The consistency of this bidirectional relationship, loss producing decreased steroidogenic output and gain producing increased output, across two independent cell line models, gives considerably more confidence in a direct causal role for BMAL1 than either knockdown or overexpression data would provide in isolation [40].

Findings from murine models extend this human cell-line evidence into an intact physiological system where circulating hormone levels, luteal function, and the full complexity of paracrine ovarian signaling remain in place. Ovarian-targeted Bmal1 deprivation in mice produces notably impaired luteal hormone synthesis, with reduced expression of star, Hsd3β2, and cyp19a1 in granulosa cells and of Lhcgr, star, Hsd3β2, and cyp17a1 in theca cells, mirroring the reduction seen in the human cell-line knockdown experiments but now demonstrated across both major steroidogenic cell compartments of the follicle rather than granulosa cells alone [41]. The mouse data additionally reveal a signaling mechanism not visible in the simpler cell-line system: Bmal1 deprivation activates phosphorylation of the PI3K/NFκB pathway, and pharmacological inhibition of PI3K using LY294002 partially rescues Lhcgr and Hsd3β2 expression in Bmal1-interfered theca cells, along with restoring androstenedione and testosterone synthesis [41]. This finding indicates that BMAL1 loss does not simply remove a positive transcriptional input from steroidogenic gene promoters but actively engages an inhibitory signaling cascade through PI3K/NFκB activation, adding a second, actively repressive mechanism operating alongside the loss of direct positive regulation, and offering a specific molecular target, PI3K inhibition, through which the downstream consequences of circadian disruption might in principle be pharmacologically addressed independent of restoring BMAL1 rhythmicity itself.

Taken together, the granulosa and theca cell evidence drawn from human cell-line knockdown/overexpression and murine ovarian-targeted knockout models supports BMAL1 operating through at least two parallel mechanisms to sustain normal steroidogenic output: a direct transcriptional role supporting expression of the core enzymatic machinery from cholesterol transport through to estrogen receptor signaling, and a restraining role over an inhibitory PI3K/NFκB signaling axis that becomes disinhibited once BMAL1 rhythmicity is lost [39]. Both mechanisms converge on the same functional endpoint—namely, impaired capacity of the follicle—to generate the estradiol signal required for normal oocyte maturation and for the endocrine feedback loops governing the ovulatory cascade. However, they facilitate this through biochemically distinct routes that would not necessarily respond identically to a single therapeutic intervention [42]. This distinction becomes practically relevant when considering melatonin supplementation later in this review, since melatonin’s mechanism of action, discussed in a later section, operates primarily through antioxidant and receptor-mediated pathways rather than through direct restoration of BMAL1 transcriptional output, meaning its capacity to reverse the PI3K/NFκB-mediated component of BMAL1 loss specifically remains an open question rather than an established finding [43].

The obesity-specific relevance of this steroidogenic disruption follows directly from the adipokine mechanism described in the preceding section. If circulating leptin, elevated in obesity, suppresses granulosa cell BMAL1 rhythmicity through the Lepr–BMAL1 loop already discussed, then the steroidogenic consequences documented here, reduced CYP11A1, CYP19A1, STAR, and ESR2 expression together with disinhibited PI3K/NFκB signaling, would be expected to manifest specifically in the ovaries of obese women undergoing ovarian stimulation, independent of any confounding effect of gonadotropin dosing or stimulation protocol [32]. This offers one specific, testable molecular prediction arising from the broader model developed in this review: granulosa cells retrieved from obese patients during oocyte pickup should show measurably reduced BMAL1 expression relative to normal-weight patients undergoing matched stimulation protocols. This comparison, to the best of available evidence, has not yet been performed directly and represents a clear opportunity for future clinical–translational work bridging the mechanistic and clinical literature, which are reviewed separately throughout this paper.

### 4.2. The BMAL1-SIRT1 Loop and Its Relevance to Polycystic Ovary Syndrome

A distinct but mechanistically related circadian pathway converges on estrogen synthesis in granulosa cells through interaction between BMAL1 and SIRT1, a NAD^+^-dependent deacetylase whose activity is itself subject to circadian and metabolic regulation, creating a second point of intersection between systemic metabolic state and local ovarian clock function beyond the leptin-mediated route already described [44]. SIRT1 has been characterized as playing a positive regulatory role in granulosa cell estrogen synthesis, and its activity and expression have been shown to interact with BMAL1 in a manner best described as a positive feedback cycle rather than a simple linear upstream–downstream relationship, with BMAL1 supporting SIRT1 expression and SIRT1 activity in turn supporting BMAL1-driven transcriptional output [44].

The relevance of this loop extends beyond obesity-related infertility narrowly defined and into polycystic ovary syndrome specifically, a condition whose pathophysiology overlaps substantially with obesity-related metabolic dysfunction, but which carries its own distinct hyperandrogenic and anovulatory features. The same study that identified this cycle also implicated its disruption in PCOS pathophysiology, proposing a contribution to the abnormal estrogen synthesis pattern characteristic of the condition [44]. This claim rests on one human granulosa-cell dataset, though, and has not yet been replicated independently. A substantial share of women with PCOS are also overweight or obese. Insulin resistance is a near-universal feature of the PCOS metabolic phenotype, and it is itself sensitive to circadian disruption [45]. On that basis, the BMAL1-SIRT1 pathway offers a plausible point of convergence between general obesity-related granulosa cell dysfunction and the more specific hormonal derangements of PCOS. No study has tested this convergence directly, though. It remains an inference built from two separate bodies of literature, rather than a demonstrated shared pathway.

This convergence matters for how the present review’s central model should be interpreted going forward. Rather than proposing obesity and PCOS as producing entirely separate downstream ovarian phenotypes through independent mechanisms, the BMAL1–SIRT1 evidence suggests both conditions may converge on a shared final pathway of granulosa cell circadian and steroidogenic dysfunction. This is a working hypothesis, not a settled conclusion. It rests on a single in vitro dataset and a plausibility argument about shared risk factors, not on any study that measured both conditions side by side. No published study has measured BMAL1 or SIRT1 in granulosa cells across simple obesity, simple PCOS, and obesity combined with PCOS as three separate groups. Zhang et al. compared PCOS against non-PCOS controls only, without stratifying either group by body weight [44]. Whether obesity and hyperandrogenism damage the granulosa cell clock through the same route, or add to each other through separate routes, has not been tested directly in human tissue. A rat model of hyperandrogenism found that excess androgen alone suppresses BMAL1 and its downstream effect on SIRT1-related signaling, independent of any obesity manipulation, which at least makes a synergistic effect plausible. That finding comes from liver and adipose tissue in rats, though, not human granulosa cells, and it should not be read as confirming what happens when obesity and PCOS occur together in a woman undergoing IVF. A three-group comparison in human granulosa cells—simple obesity, simple PCOS, and both together is the direct test this section is missing and remains open for future work. This has a direct bearing on how melatonin supplementation trials, several of which have specifically enrolled PCOS populations undergoing IVF, should be interpreted within the broader framework developed throughout this review, a point returned to explicitly when the clinical melatonin literature is addressed later in this manuscript [44].

### 4.3. Mitochondrial Biogenesis, the Unfolded Protein Response, and Oocyte Quality

Steroidogenic disruption represents only one consequence of granulosa cell BMAL1 loss; a second and arguably more consequential pathway operates through mitochondrial function, given that oocyte developmental competence depends critically on adequate mitochondrial mass, membrane potential, and ATP-generating capacity supplied in large part through paracrine and gap-junction communication with the surrounding granulosa and cumulus cell compartment [46]. Granulosa cell mitochondria are not incidental bystanders to steroidogenesis but functionally integrated with it, since the rate-limiting cholesterol side-chain cleavage step catalyzed by CYP11A1 occurs specifically within the mitochondrial inner membrane, meaning mitochondrial health and steroidogenic capacity are mechanistically inseparable rather than parallel independent processes that happen to be affected together [46].

Mitochondrial dysfunction in human granulosa cells correlates directly with declining steroidogenesis, reduced oocyte maturation rate, lower fertilization rate, and diminished oocyte quality across patient groups, including those with endometriosis, ovarian endometrioma, and PCOS, establishing mitochondrial competence as a shared final pathway across several distinct causes of subfertility rather than a mechanism specific to any single diagnosis [47]. Granulosa cell mitochondrial number, ultrastructural integrity, and membrane potential have each been proposed as candidate biomarkers of oocyte competence precisely because of this tight coupling between the somatic support cell compartment and the oocyte’s own developmental trajectory, reflecting the reality that the oocyte itself possesses relatively limited independent capacity for mitochondrial biogenesis during the final stages of maturation and depends substantially on cumulus and granulosa cell metabolic support delivered through transzonal projections [48].

Circadian regulation intersects this mitochondrial biology at the level of biogenesis itself, since BMAL1 and its downstream targets participate in coordinating mitochondrial DNA replication and the transcription of nuclear-encoded mitochondrial proteins in a time-of-day-dependent manner across multiple tissue types, a relationship well established in the liver and skeletal muscle and increasingly recognized in reproductive tissue [49]. Loss of BMAL1 rhythmicity would therefore be expected to desynchronize mitochondrial biogenesis from the metabolic demand cycles it normally anticipates, producing a mismatch between mitochondrial capacity and the energetic requirements of oocyte maturation at any given point in the stimulation cycle, though direct measurement of this specific desynchronization in obese granulosa cells during controlled ovarian stimulation remains an area requiring further targeted investigation rather than one already comprehensively characterized in the existing literature. Most of what is known about BMAL1 and mitochondrial biogenesis comes from liver and muscle, not the ovary. The ovarian extension argued here is a reasonable inference from that other-tissue biology. It is not yet a finding in its own right.

The unfolded protein response operating specifically within the mitochondrion, denoted UPRmt, provides an additional layer of quality control relevant to this discussion, activated when misfolded or unassembled mitochondrial proteins accumulate beyond the chaperone system’s capacity to manage them. Under conditions of chronic circadian disruption, the demand supply mismatch just described would be expected to increase the burden of misfolded mitochondrial protein, engaging UPRmt as a compensatory response, and chronic UPRmt activation, if sustained rather than transient. Is generally associated with declining rather than improving cellular function across multiple tissue systems characterized in the broader mitochondrial stress literature, offering a plausible route by which circadian disruption in obesity could translate into cumulative granulosa cell dysfunction over the course of repeated stimulation cycles rather than manifesting as an acute, single-cycle effect alone.

### 4.4. HIF-1α/BNIP3-Mediated Mitophagy During Luteinization

A related but mechanistically distinct pathway concerns mitophagy, the selective autophagic clearance of damaged or superfluous mitochondria, which plays an active and physiologically necessary role during the luteinization process itself rather than functioning purely as a pathological response to injury [50]. As granulosa cells transition into luteinized cells following the LH surge, HIF-1α/BNIP3-mediated autophagy has been shown to contribute directly and necessarily to normal luteinization, with markers of autophagic activity, including LC3-I/II conversion and Beclin1 expression, increasing specifically during this transition and correlating with successful formation of the corpus luteum [51].

This finding carries an important implication that runs somewhat counter to an oversimplified reading of mitochondrial dysfunction as uniformly harmful: some degree of mitophagic turnover during luteinization appears to be a required physiological process rather than an unwanted consequence of cellular stress. On this notion, the relevant question for circadian disruption is not simply whether mitophagy occurs, but whether its timing and magnitude remain appropriately calibrated to the luteinization process [52]. Given that circadian clock genes have been shown to regulate autophagy-related gene expression directly in other reproductive contexts, including NR1D1-mediated repression of autophagy-related transcription in granulosa cells documented in the sleep-disruption literature discussed in the Introduction, it is plausible that BMAL1 loss disrupts not merely the presence or absence of mitophagy during luteinization but its precise circadian timing relative to the LH surge and subsequent luteal transition, producing either premature or delayed mitochondrial clearance relative to the cell’s actual physiological need at that point in the luteinization sequence [53].

### 4.5. Oxidative Stress, Oocyte DNA Damage, and the Meiotic Spindle

The final convergent pathway linking granulosa cell circadian disruption to impaired oocyte competence operates through oxidative stress a mechanism with the most direct existing clinical corroboration of any discussed in this section, given the substantial melatonin supplementation literature addressed later that specifically targets oxidative pathways. Intrafollicular concentrations of 8-hydroxy-2′-deoxyguanosine, a well-established marker of oxidative DNA damage, are measurably elevated in the follicular fluid of women with high rates of degenerate oocytes. Relative to those with low rates, this elevation correlates inversely and specifically with intrafollicular melatonin concentration, establishing oxidative damage and antioxidant capacity as directly opposed and quantifiable forces operating within the same follicular microenvironment [9].

Mitochondrial dysfunction of the kind described in the preceding two subsections represents a major intracellular source of reactive oxygen species, since impaired electron transport chain function characteristically increases electron leakage and consequent superoxide generation rather than simply reducing ATP output alone [54]. Circadian disruption of granulosa cell mitochondrial biogenesis and mitophagy, as detailed above, would therefore be expected to increase local oxidative burden through this mitochondrial route specifically, independent of any additional oxidative contribution arising from the systemic inflammatory state characteristic of obesity and already discussed in the preceding section on adipose tissue [55]. Oocytes exposed to this combined oxidative burden, arising from both mitochondrial dysfunction locally within the follicle and inflammatory cytokine exposure arriving systemically, show disrupted meiotic spindle formation and altered mitochondrial dynamics within the oocyte’s own cytoplasm, translating the granulosa cell-level dysfunction described throughout this section into a direct, structurally demonstrable consequence within the oocyte itself [56].

The convergence of multiple mechanistically distinct pathways, direct steroidogenic transcriptional disruption, PI3K/NFκB disinhibition, mitochondrial biogenesis desynchronization, dysregulated UPRmt activation, and mistimed mitophagy, all ultimately funneling into a shared oxidative stress endpoint, offers a coherent account of why granulosa cell BMAL1 loss produces oocyte quality deficits substantial enough to be clinically detectable despite arising from what is, at the transcriptional level, a relatively modest and gradual amplitude reduction rather than a complete loss of clock function. Multiple partially redundant pathways converging on a single oxidative endpoint would be expected to produce a more robust and less easily compensated phenotype than any single pathway acting in isolation, which may help explain why oocyte quality deficits in obesity have proven so consistently reproducible across studies despite the mechanistic complexity and apparent redundancy involved. The endometrial consequences of the same underlying BMAL1 disruption, operating through parallel but tissue-specific mechanisms in the uterine compartment, are addressed directly in the section that follows.

## 5. Molecular Mechanisms in Endometrial Clock Disruption

### 5.1. BMAL1 in Decidualization and the Control of Trophoblast Invasion

The endometrium carries its own autonomous circadian clock, distinct in both location and function from the ovarian oscillator described in the preceding section yet built on the identical BMAL1: CLOCK transcriptional core. Human endometrial stromal cells, both immortalized lines and primary cultures, show measurable BMAL1 expression that changes substantially upon induction of decidualization in vitro, with BMAL1 levels rising as stromal cells transition into the decidualized phenotype required to support trophoblast invasion and early pregnancy maintenance [8]. This upregulation during decidualization suggested from the outset that BMAL1 was not merely present in endometrial tissue as an incidental feature of having nucleated cells with a general transcriptional apparatus, but functionally engaged in the differentiation program itself, a hypothesis subsequently confirmed through direct loss-of-function experiments.

Silencing BMAL1 in decidualizing endometrial stromal cells produces impaired decidualization, evident both morphologically and at the level of specific molecular markers. IGFBP1 and prolactin, the two most widely used biochemical readouts of successful stromal decidualization, both show reduced expression following BMAL1 knockdown, indicating that the differentiation program itself fails to complete normally in the absence of adequate BMAL1 signaling rather than proceeding to completion with only cosmetic molecular differences [57]. Beyond this general decidualization deficit, BMAL1 silencing produces a specific and mechanistically informative downstream consequence: reduced expression of TIMP3, a tissue inhibitor of metalloproteinases that ordinarily restrains the proteolytic activity permitting trophoblast cells to invade the maternal decidua during implantation [8].

The functional consequence of reduced TIMP3 is not a failure of invasion but its opposite, an overinvasion phenotype in which trophoblast cells penetrate the decidual compartment more extensively than is compatible with normal placental development [58]. This finding reframes what BMAL1 loss produces at the maternal-fetal interface, since the intuitive expectation, given that BMAL1 loss elsewhere in this review has generally been associated with reduced function, might be that endometrial BMAL1 loss would similarly reduce trophoblast invasion and produce a failure of implantation through insufficient rather than excessive invasive capacity [59]. The TIMP3 mechanism demonstrates instead that BMAL1 operates in this tissue as a brake on a process that requires careful calibration rather than simple maximization, with either insufficient or excessive invasion representing a departure from the narrow physiological range compatible with successful pregnancy, and BMAL1 loss specifically removing restraint rather than removing drive [60].

Clinical correlation supports the physiological relevance of this mechanism beyond the cell-culture system in which it was first characterized. BMAL1 expression is measurably reduced in decidual tissue obtained from women with recurrent miscarriage relative to women without this history, establishing an association between endometrial clock disruption and a clinically significant adverse pregnancy outcome rather than leaving the mechanism as an isolated in vitro observation without apparent clinical counterpart [8]. Genetic evidence reinforces this association independently of the expression-level findings. Single-nucleotide polymorphism analysis has identified the BMAL1 rs2278749 TT genotype as associated with increased miscarriage risk, indicating that constitutional variation in BMAL1 function present from conception rather than arising as an acquired consequence of obesity or other environmental exposure carries reproductive consequences consistent with the direction of acquired disruption described throughout this review [61].

The relevance of this mechanism to the obesity-centered model developed in this paper follows from the adipokine and inflammatory routes established in the preceding sections. If circulating leptin and inflammatory cytokines, elevated systemically in obesity through the mechanisms detailed earlier, reach endometrial stromal cells and suppress BMAL1 expression there through pathways analogous to those already demonstrated in granulosa cells. Then, the TIMP3-mediated overinvasion phenotype described here would be predicted to occur with elevated frequency in obese women undergoing embryo transfer, offering one specific mechanistic candidate for the endometrial contribution. Reducing IVF success in this population, a contribution kept mechanistically distinct from, though potentially co-occurring with, the granulosa cell and oocyte-level disruption addressed in the previous section.

### 5.2. PER2 and the Silencing of Circadian Oscillation During Decidual Transformation

A related but mechanistically distinct line of evidence concerns PER2, the negative-arm clock component whose behavior during decidualization reveals something unexpected about how the endometrial clock handles the transition from a cycling, undifferentiated stromal cell to a terminally decidualized one [62]. Undifferentiated human endometrial stromal cells, when synchronized in culture, show clear circadian oscillation of the core clock genes, CLOCK, BMAL1 (ARNTL), CRY1, CRY2, PER1, and PER2, with amplitude varying up to fivefold across a twenty-six-hour period, confirming that undifferentiated stromal cells carry a fully functional autonomous oscillator comparable in robustness to that described in adipose tissue and granulosa cells elsewhere in this review [62].

What happens upon induction of decidualization departs from what might be predicted by analogy with the granulosa cell and adipocyte systems already discussed, where disruption of rhythmicity has consistently been associated with pathological consequences [63]. In decidualizing stromal cell cultures, expression of all six core clock genes becomes uniformly aperiodic, confirming that terminal differentiation into the decidualized phenotype is itself accompanied by a genuine silencing of circadian oscillation rather than merely a shift in its phase or amplitude [62]. This loss of rhythmicity is not evidence of pathological clock failure in this specific context but appears to represent a deliberate and physiologically appropriate feature of the differentiation program, since the mechanism responsible has been identified with some precision: PER2 expression is lost specifically because of attenuated binding of the CLOCK transcription factor complex to the E2 enhancer element within the proximal PER2 promoter. Occurring during decidualization despite the CLOCK/BMAL1 heterodimer’s DNA-binding activity remaining intact at other clock gene promoters, including that of PER1, as confirmed through chromatin immunoprecipitation analysis [62].

This selective, promoter-specific loss of CLOCK binding at PER2 specifically, without a general collapse of CLOCK/BMAL1 DNA-binding capacity across the genome, indicates that decidualization actively and selectively remodels the clock’s target gene network rather than simply overwhelming or degrading the transcriptional machinery wholesale [64]. The distinction matters considerably for how obesity-related endometrial clock disruption, as opposed to the physiological silencing described here, should be conceptualized. What decidualizing cells appear to achieve deliberately in a controlled, promoter-specific manner as part of normal differentiation obesity may impose in a dysregulated and untimed fashion, thereby disrupting clock function before decidualization has been appropriately triggered or independent of the differentiation signal altogether. This produces a pathological rather than physiological loss of rhythmicity that arrives at the wrong time and through the wrong mechanism relative to the tissue’s actual developmental state [65].

This distinction between a controlled clock silencing coordinating mitotic expansion and decidual transformation and an obesity-driven clock disruption imposed independently of the tissue’s differentiation status has not to date been directly tested within a single experimental system. Conducting such a comparison would require comparing endometrial stromal cells from obese and lean women at matched stages of the decidualization process rather than relying on separate cell-culture systems from which the current evidence is drawn. Establishing whether obesity accelerates, delays, or simply desynchronizes the PER2 silencing process described here from its normal coupling to decidualization onset represents a specific and currently unaddressed question that follows directly from combining the mechanistic threads reviewed in this subsection with the broader obesity-centered framework developed throughout this paper.

### 5.3. The BMAL1/REV-ERB Loop and Growth Factor Signaling During Implantation

A further layer of endometrial clock function operates through the transcriptional relationship between BMAL1 and REV-ERB acting jointly on growth factor genes directly implicated in the mechanics of embryo implantation rather than in decidualization considered narrowly. BMAL1 transcriptionally activates REV-ERB expression, and REV-ERB in turn binds RORE motifs present in the promoters of Bmp2, Bmp4, Gdf10, and Gdf15, forming a feedback arrangement that couples the core clock oscillation to the transcriptional dynamics of these specific growth factors within uterine endometrial stromal cells [66].

BMP2 and BMP4 together with GDF10 and GDF15 occupy a well-established position within the implantation cascade, contributing to stromal decidualization and to the coordination of trophoblast-stromal signaling required for successful placental development, functions that sit adjacent to but mechanistically distinct from the TIMP3-mediated invasion control described in the preceding subsection [67]. The BMAL1/REV-ERB loop therefore constitutes a second, parallel route by which circadian disruption reaches the implantation process, operating through growth factor transcriptional dynamics rather than through the protease-inhibitor balance governing trophoblast invasion depth. The existence of two mechanistically separate circadian inputs converging on implantation success offers a partial explanation for why implantation failure in the clinical literature proves so heterogeneous in its apparent causes, since disruption entering through either route, or through both simultaneously, could plausibly produce a broadly similar clinical phenotype despite arising from distinct underlying molecular lesions [68].

Circadian misalignment more broadly, beyond the specific BMAL1/REV-ERB/growth factor axis just described, has been shown to disrupt implantation through additional convergent pathways operating at the level of local immune and inflammatory signaling within the endometrium [66]. Macrophage-derived interleukin-6 and leukemia inhibitory factor, together with endometrial epithelial cell-secreted COX-2, are each required for normal blastocyst adhesion during the initial receptivity stage of implantation, and circadian disruption has been implicated in altering the timing and magnitude of their local secretion [66]. A separate systemic route operates through disrupted rhythmic secretion of cortisol, insulin, and ghrelin, hormones whose normal circadian patterning depends on intact central and peripheral clock coordination, and whose disrupted rhythms, once reaching the endometrium via systemic circulation, further impair the decidual transformation and trophoblast function processes already discussed as governed locally by BMAL1 and REV-ERB.

The layering of local transcriptional disruption, local immune signaling disruption, and systemic hormonal disruption, all converging on the same implantation process from different mechanistic directions, mirrors the pattern already established in the granulosa cell section of this review, where multiple partially independent pathways were similarly shown to funnel toward a shared oxidative stress endpoint. The recurrence of this convergent, multi-pathway architecture across both the ovarian and endometrial compartments considered separately in this review suggests it may represent a general feature of how circadian disruption produces reproductive dysfunction, rather than a coincidental similarity between two otherwise unrelated tissue systems, an observation returned to explicitly in the Discussion when the overall coherence of the model proposed in this paper is assessed.

### 5.4. HOXA10, Displacement of the Window of Implantation, and Convergence with Metabolic Signaling

HOXA10 occupies a well-established position in the endometrial receptivity literature independent of any circadian consideration, functioning as a homeobox transcription factor required for normal endometrial differentiation during the window of implantation, with reduced HOXA10 expression previously associated with unexplained infertility and recurrent implantation failure in contexts entirely unrelated to circadian biology [69]. The relevance of HOXA10 to the present discussion arises from evidence that its expression is itself influenced by light–dark cycle manipulation, with animal studies showing that extended light exposure regimens reduce HOXA10 expression and increase the frequency of uterine structural abnormalities relative to standard light dark cycling, and continuous light exposure producing comparable effects in independent experiments [70].

The precise molecular mechanism connecting specific core clock genes to HOXA10 transcription has not yet been fully characterized, representing one of the more clearly acknowledged gaps in the current literature reviewed here rather than a fully resolved pathway comparable to the BMAL1-TIMP3 or BMAL1/REV-ERB growth factor mechanisms detailed in the preceding subsections [71]. What is established, however, is that melatonin, itself both an output and a modulator of the core clock system discussed extensively in a later section of this review, upregulates CLOCK and PER1 expression and independently promotes HOXA10 expression in endometrial tissue, suggesting an indirect route by which circadian signaling reaches HOXA10 transcription even without full mechanistic delineation of the specific intermediate steps involved [72].

The clinical phenomenon most directly relevant to this discussion, and to the obesity-centered framework developed throughout this review, is displacement of the window of implantation, documented directly through endometrial receptivity array analysis in obese women relative to normal-weight controls. A prospective study using this technology found displacement of the window of implantation present in 25.3 percent of obese women compared with 9.7 percent of non-obese women, with the magnitude of displacement increasing progressively with higher obesity class. This fits a dose-dependent relationship between adiposity and receptivity, not a simple present-or-absent effect. The study did not measure BMAL1 directly, though. The link to the circadian mechanism proposed here stays inferential [3]. This displacement phenomenon offers perhaps the clearest existing clinical bridge between the molecular mechanisms detailed throughout this section and the donor-oocyte clinical literature addressed in the section that follows, since a displaced window of implantation would be expected to reduce implantation success specifically through an endometrial-timing mechanism, independent of oocyte quality, precisely the kind of endometrium-specific effect that the donor-oocyte model was designed to isolate.

Convergence with the systemic metabolic signals introduced earlier in this section, cortisol, insulin, and ghrelin, becomes particularly relevant when considering why the window of implantation specifically, rather than decidualization capacity or trophoblast invasion control considered independently, might prove most sensitive to obesity-related disruption among the several endometrial processes discussed in this section [73]. The window of implantation represents a narrowly time-limited period requiring precise synchronization between endometrial receptivity and blastocyst developmental stage, and a process depending this critically on exact timing would be expected to show disproportionate vulnerability to any input that degrades the precision of the underlying circadian signal, relative to processes such as decidualization capacity that while still circadian-influenced according to the evidence reviewed in the preceding two subsections do not depend on the same narrow temporal window for their successful completion [74]. This timing-sensitivity argument offers one plausible explanation for why the window of implantation displacement has emerged as such a consistent and quantifiable finding in the obesity IVF literature specifically, relative to the somewhat more variable and less consistently reproduced effects of obesity on decidualization capacity or trophoblast invasion considered as isolated endpoints, a pattern that the clinical literature addressed in the next section of this review will be examined against directly [75]. Before turning to the clinical literature testing these mechanisms directly, Table 1 summarizes the molecular pathways described across the preceding three sections, organized by tissue compartment to make the recurring pattern of convergent, partially redundant mechanisms easier to follow.

## 6. Clinical Correlates: The Donor-Oocyte Model as a Natural Experiment

### 6.1. The Logic of the Donor-Oocyte Model and Its Interpretive Value

Separating an oocyte-level effect of obesity from an endometrium-level effect has proven persistently difficult using conventional autologous IVF cohorts, since obese women undergoing IVF with their own oocytes present both potential sources of impairment simultaneously, leaving no straightforward way to attribute a reduced success rate to one reproductive compartment rather than the other [77]. Fertilization rate, blastulation rate, and implantation rate are each influenced by oocyte quality, and any observed reduction in one of these parameters in an obese autologous cohort could in principle originate from impaired oocyte competence, impaired endometrial receptivity, or some combination of the two acting jointly, without the study design itself offering any means of partitioning the contribution of each [78].

The donor-oocyte model resolves this ambiguity through a deliberately constructed natural experiment rather than through statistical adjustment for confounders after the fact. Recipients receive oocytes retrieved from young, healthy, normal-weight donors selected specifically to minimize variability in oocyte quality across the cohort, meaning any variation in downstream implantation or pregnancy outcome according to the recipient’s own BMI can be attributed with considerably greater confidence to the recipient’s endometrial environment, since the oocyte itself carries none of the recipient’s own metabolic history, adipokine exposure, or systemic inflammatory burden [79]. The model is not without limitations of its own, since donor stimulation protocols, donor age range, and laboratory handling of donated oocytes introduce their own sources of variability across studies and across clinics, and these methodological differences complicate direct comparison between cohorts in ways addressed further in the subsections that follow.

A further confound belongs here, and it concerns the recipient rather than the donor. Autologous IVF and donor-oocyte cycles prepare the endometrium in fundamentally different ways. In autologous IVF, the endometrium develops under the hormonal milieu generated by the patient’s own stimulated cycle. In donor-oocyte cycles, the recipient’s ovaries are typically suppressed, and the endometrium is instead built through a programmed regimen of exogenous estradiol and progesterone, timed independently of any endogenous follicular development [79]. This distinction is rarely made explicit in the studies discussed below. A lower implantation rate in obese recipients could in principle reflect differences in exogenous hormone dosing or absorption under a fixed protocol, rather than the endometrial circadian mechanism proposed in this review. None of the donor-oocyte studies discussed in the following subsections report whether estradiol dosing was individualized or adjusted by recipient body weight, and this gap limits how confidently any of them can rule out a non-circadian, protocol-related explanation for a recipient BMI effect.

What makes this body of literature particularly valuable for the purposes of the present review is that it constitutes the most direct available clinical test of the endometrial circadian mechanisms detailed in the preceding section, specifically the TIMP3-mediated control of trophoblast invasion depth, the BMAL1/REV-ERB transcriptional loop governing BMP and GDF growth factor expression, and the demonstrated displacement of the transcriptional window of implantation in obesity [80]. If these circadian mechanisms operate as proposed, and if their severity scales with the degree of systemic adiposity through the adipokine and inflammatory routes established earlier in this review. Then, the donor-oocyte cohorts should reveal a negative association between recipient BMI and implantation success that becomes progressively more pronounced as the sample size and obesity-class range increase. This is because larger and more heterogeneous cohorts would be expected to capture the full dose response relationship, rather than being restricted to a narrow band of BMI values where any true effect might remain below the threshold of statistical detectability [79].

The donor-oocyte literature as it currently stands does not speak with one voice on this question, and the interpretive task facing anyone attempting to draw conclusions from it is complicated by the fact that individual studies vary considerably in sample size, in the BMI thresholds used to define comparison groups, in whether embryo ploidy status was controlled for directly, and in whether oocytes were split between recipients from a single donor cycle or drawn independently across separate donation cycles [81]. Each of these design choices carries implications for statistical power and for the specific mechanistic question that a given study is equipped to answer. Meanwhile, reading the literature as a simple binary contest between studies finding an effect and studies finding none risks obscuring these more substantive differences in what each study was designed and powered to detect. The two subsections that follow present the null-finding and the positive-finding literature, respectively. They are then followed by a final subsection that considers what methodological and biological factors might account for their divergence in light of the circadian mechanisms proposed throughout this review. This is an exercise to reconcile rather than to simply declare one body of evidence as correct and the other mistaken [82].

Another consideration bearing on how this literature should be read concerns publication timing and the evolution of laboratory practice across the roughly two decades this body of work spans. Embryo culture conditions, vitrification technique, and endometrial preparation protocols have each changed substantially between the earliest donor-oocyte cohorts discussed below and the most recent, and any of these technical evolutions could in principle interact with recipient BMI in ways not explicitly modeled in the original studies, since a laboratory or clinical protocol optimized under conditions prevailing at one point in time need not perform identically across the full range of recipient metabolic phenotypes represented in a given cohort [83]. This temporal heterogeneity does not invalidate the comparisons drawn in the subsections that follow, but it argues for some caution in treating the donor-oocyte literature as a single homogeneous body of evidence accumulated under constant methodological conditions, rather than as a literature that has itself evolved alongside the clinical practice of assisted reproduction over the period it covers. Table 2 outlines the two competing explanatory frameworks this model is designed to arbitrate between, together with the specific pattern of donor-oocyte evidence each would predict, before the individual studies are examined in the subsections that follow.

### 6.2. Studies Reporting No Effects of Recipient BMI on Implantation

Several methodologically distinct donor-oocyte cohorts converge on the conclusion that recipient BMI carries no measurable association with implantation outcome, and the consistency of this null finding across studies employing somewhat different designs deserves consideration in its own right rather than being dismissed simply as a series of underpowered negative results [84]. An early retrospective analysis drawing on five hundred thirty-six first-cycle donor oocyte recipients divided across four BMI categories spanning underweight through obese found no adverse effect of recipient BMI on implantation rate, pregnancy rate, or incidence of spontaneous miscarriage [4]. This study’s methodological strength lay in its comprehensive stratification across the full BMI range rather than a simple obese-versus-non-obese binary comparison, meaning a genuine effect concentrated specifically at either extreme of the distribution should in principle have been detectable even without a formal dose–response analysis.

A subsequent single-center study addressed a specific confound not directly controlled for in the earliest cohorts, namely embryo ploidy status, by restricting analysis exclusively to transfers of euploid embryos following preimplantation genetic testing, thereby removing chromosomal abnormality as a potential source of variation that might otherwise obscure or mimic a BMI-related effect. Clinical pregnancy rate, live birth rate, biochemical pregnancy loss rate, and clinical pregnancy loss rate each showed no significant difference across normal-weight, overweight, and obese recipient groups within this euploid-restricted cohort [85]. The absence of an effect even after controlling for ploidy status strengthens the null finding considerably relative to earlier studies lacking this genetic confirmation, since it rules out the possibility that a BMI-associated difference in aneuploidy rate among transferred embryos, rather than a true endometrial receptivity effect, might have been masking or mimicking a genuine relationship in the earlier, ploidy-unconfirmed cohorts.

The sibling-oocyte paired design reported by Setton and colleagues offers what is arguably the single most tightly controlled test available within this literature, since the two recipients compared within each pair received oocytes retrieved from an identical donor during one shared stimulation cycle, eliminating not only oocyte quality broadly construed but also any donor-specific or cycle-specific variation in oocyte cohort characteristics that could persist even across separate donation cycles involving equally young and healthy donors. Implantation rates proved statistically indistinguishable between the normal-weight and overweight-or-obese members of these matched pairs, a finding that carries particular interpretive weight precisely because the paired design controls for a source of variability, inter-donor and inter-cycle heterogeneity, that none of the unpaired cohort studies discussed above were structurally capable of addressing [84].

An earlier study employing a related logic, comparing BMI against uterine receptivity using receiver operating characteristic curve analysis rather than a simple stratified comparison, likewise found no relationship between recipient BMI and implantation outcome, with an area under the curve close to the null value of 0.5, corresponding to essentially no discriminative capacity of BMI to predict implantation success in that particular cohort [86]. Taken as a group, these four studies span a range of design strategies, unstratified cohort comparison, ploidy-restricted comparison, sibling-paired comparison, and ROC-based discrimination analysis, yet converge on a consistent absence of detectable BMI effect, a convergence across methodologically distinct approaches that argues against dismissing the null finding as an artifact specific to any single study design.

### 6.3. Studies Reporting Negative Effects of Recipient BMI on Implantation

Set against this consistent body of null findings, the largest single-center donor-oocyte series published to date reaches a markedly different and considerably more concerning conclusion regarding the reproductive consequences of recipient obesity. Analysis of 9587 first cycles of ovum donation, drawing exclusively on oocytes retrieved from normal-weight donors across a twelve-year period, found implantation rate, clinical pregnancy rate, and live-birth rate each declining in a stepwise fashion across four recipient BMI categories, from lean through normal weight, overweight, and obese, with live-birth rate falling from 38.6 percent among lean recipients to 27.7 percent among obese recipients [3]. The magnitude of this decline, more than ten percentage points in absolute live-birth rate between the extremes of the BMI distribution, represents a clinically substantial effect size rather than a marginal statistical difference in uncertain practical relevance.

A detail of considerable mechanistic importance in this same cohort is that in vitro fertilization laboratory parameters, including fertilization rate and cleavage rate, did not differ across the four BMI groups, indicating that whatever process was responsible for the observed decline in live birth operated downstream of the fertilization and early cleavage stages captured by standard laboratory metrics. Since donor oocytes were used uniformly across all recipient BMI categories in this cohort, any residual embryo-quality difference attributable to the oocyte itself should have been minimized by design, leaving endometrial receptivity as the most parsimonious explanation for a decline that tracked specifically with recipient BMI despite laboratory-stage parameters remaining constant across groups.

Independent corroboration of an endometrial mechanism specifically, rather than an inference drawn indirectly from pregnancy outcome data alone, comes from direct measurement of endometrial gene expression through receptivity array testing, which demonstrated a displaced window of implantation in obese women relative to non-obese controls, with displacement present in 25.3 percent of obese women compared with 9.7 percent of non-obese women and increasing progressively across obesity classes [3]. This transcriptional evidence carries particular weight in the present context because it measures the proposed mechanism directly rather than inferring its presence from a downstream clinical outcome susceptible to numerous alternative explanations, and a demonstrated shift in the receptivity window constitutes a qualitatively different and arguably stronger form of evidence than a pregnancy-rate difference considered in isolation, however large that difference may be. Table 2 summarizes the donor-oocyte studies discussed in the two preceding subsections side by side, making the divergence in design, sample size, and obesity-class composition easier to evaluate before the reconciliation attempted below.

### 6.4. Reconciling the Discrepancy

One plausible, testable explanation for the divergence between these two bodies of evidence is that it reflects differences in statistical power and in the distribution of obesity severity represented across studies, rather than a genuine disagreement about the underlying reproductive biology at stake; this explanation is proposed as a hypothesis warranting direct testing rather than as the established resolution of the discrepancy, and alternative explanations cannot be excluded on the basis of current data. Consistent with this hypothesis, the Bellver 2013 cohort’s obese subgroup comprised 653 cycles drawn from a total sample of 9587, a subgroup size an order of magnitude larger than the entire cohorts examined in several of the null-finding studies discussed above, and correspondingly better positioned to detect a moderate effect size that smaller, otherwise carefully designed cohorts would plausibly fail to reach statistical significance on, not because the underlying effect was necessarily absent in those smaller cohorts but because their sample sizes may have been inadequate to detect it reliably [87]. Moreover, the stepwise, four-category dose–response pattern documented in the largest cohort differs structurally from the binary or tripartite BMI comparisons employed in several of the null-finding studies. Additionally, a true biological gradient distributed across four groups could easily fail to reach significance when collapsed into two broader comparison categories, even with constant total sample size.

A second and non-competing consideration concerns the specific obesity-class composition of the comparison groups across studies. Several of the null-finding cohorts, including the sibling-oocyte and euploid-embryo analyses, drew their overweight-or-obese comparison groups predominantly from the lower end of the obesity spectrum rather than from class II or III obesity specifically. In contrast, the dose–response decline documented in the largest cohort proves most pronounced precisely within these more severe obesity classes [84]. This pattern is broadly consistent with the mechanistic framework proposed throughout this review, in which adipokine signaling, inflammatory cytokine burden, and the severity of circadian disruption at the adipocyte level are expected to scale with the degree of adiposity rather than with the simple presence or absence of a BMI threshold. This implies that a genuine endometrial effect might only become clinically detectable once obesity crosses a certain severity threshold—a threshold that several of the smaller, milder-obesity-weighted cohorts may simply not have reached in sufficient numbers to characterize.

## 7. Melatonin as a Chronobiotic Modulator: Mechanistic and Clinical Evidence

### 7.1. Melatonin’s Dual Position Within the Circadian System

Melatonin occupies an unusual position within the circadian architecture detailed throughout this review, functioning simultaneously as a downstream output of the central suprachiasmatic pacemaker and as an upstream input signal capable of directly modulating peripheral clock function in target tissues, a dual role that sets it apart mechanistically from the transcriptional and epigenetic pathways described in the preceding sections [88]. Secreted by the pineal gland under tight suprachiasmatic control, its characteristic nocturnal rise provides one of the principal systemic timing cues by which peripheral oscillators throughout the body, including those in adipose tissue, granulosa cells, and endometrial stroma, remain synchronized to the external light dark cycle rather than drifting independently at their own intrinsic period [89].

Melatonin receptors of the MT1 and MT2 subtypes are expressed directly within granulosa cells and endometrial tissue, giving the hormone a route to act locally at the level of the reproductive tissues considered throughout this review rather than exerting its influence solely through resetting the central pacemaker and allowing downstream effects to propagate indirectly through altered systemic entrainment [90]. This local receptor expression matters considerably for how melatonin’s therapeutic potential should be understood, since a hormone acting exclusively through central resetting would be expected to require considerably longer exposure before producing measurable peripheral tissue effects, whereas direct local receptor engagement offers a plausible route to more rapid tissue-level action independent of any change in the broader systemic circadian phase [91].

The mechanistic distinctiveness of melatonin relative to the pathways detailed earlier in this review bears directly on how its therapeutic promise should be interpreted rather than assumed. Restoring Bmal1 promoter accessibility through glutamine or methionine supplementation, as demonstrated mechanistically in adipose tissue, addresses the specific epigenetic substrate limitation identified as the proximate cause of clock disruption in that tissue [16]. Melatonin, by contrast, does not act primarily through this same epigenetic route, operating instead through a combination of direct receptor-mediated signaling and independent free-radical scavenging capacity, discussed in detail in the following subsection, without necessarily correcting the upstream transcriptional lesion responsible for degraded BMAL1 expression in the first place [92]. This distinction carries a practical implication worth stating plainly before considering the clinical evidence: melatonin supplementation might plausibly compensate functionally for some downstream consequences of reduced BMAL1 rhythmicity, such as oxidative burden in the follicular compartment. Without restoring BMAL1 transcription itself to its normal amplitude, meaning clinical benefit observed in supplementation trials need not indicate that the underlying circadian lesion described mechanistically throughout this review has been corrected, only that one or more of its downstream physiological consequences have been mitigated through a parallel pathway [93].

Whether melatonin might additionally influence BMAL1 expression directly, beyond its established antioxidant and receptor-mediated actions, remains a question indirectly addressed by the existing literature. Some evidence discussed in the endometrial section of this review indicates that melatonin upregulates CLOCK and PER1 expression in endometrial tissue, suggesting at least some capacity to influence core clock transcription rather than acting purely downstream of it, though whether an equivalent direct transcriptional effect on BMAL1 itself occurs in granulosa cells or adipose tissue has not been established with comparable specificity [66]. This uncertainty regarding melatonin’s precise mechanistic reach, whether it operates purely downstream of BMAL1 disruption or additionally feeds back to influence BMAL1 transcription itself, is worth holding in mind through the clinical evidence presented in the remainder of this section, since the two possibilities carry rather different implications for how completely melatonin supplementation might be expected to address the obesity-driven mechanisms detailed earlier in this review.

### 7.2. Antioxidant Mechanism in the Follicular Compartment

Melatonin’s best-characterized and most mechanistically direct action in reproductive tissue operates through free-radical scavenging combined with upregulation of endogenous antioxidant enzyme systems, a property first established through direct measurement of intrafollicular melatonin concentration and its inverse relationship with 8-hydroxy-2′-deoxyguanosine, the oxidative DNA damage marker whose elevation in degenerate oocytes was introduced earlier in this review in the context of granulosa cell mitochondrial dysfunction. Administration of oral melatonin at 3 mg per day to women undergoing IVF-embryo transfer measurably raised intrafollicular melatonin concentration and correspondingly reduced this oxidative damage marker, with a parallel improvement in fertilization rate observed in the same patient population [9].

The follicle appears to concentrate melatonin considerably above circulating serum levels even in the absence of exogenous supplementation, a property attributed to active local accumulation by granulosa cells rather than simple passive diffusion from the systemic circulation [94]. This concentrating capacity plausibly explains why a modest oral dose achieves a follicular effect substantial enough to influence measurable oxidative and fertilization outcomes, despite the relatively low bioavailability characteristic of orally administered melatonin more generally. Local granulosa cell melatonin synthesis has additionally been proposed as contributing to this follicular concentration independent of exogenous or pineal-derived melatonin reaching the follicle through the circulation, meaning the follicular melatonin pool available to protect the oocyte during its final maturation stages may reflect a combination of local synthesis, active concentration of circulating hormone, and any exogenous supplementation superimposed on top of both [95].

The relevance of this antioxidant mechanism to the obesity-centered framework developed throughout this review follows directly from the convergent oxidative stress pathway identified earlier as the shared endpoint of multiple mechanistically distinct forms of granulosa cell circadian disruption, spanning mitochondrial biogenesis desynchronization, dysregulated UPRmt activation, and mistimed mitophagy during luteinization [96]. If obesity increases oxidative burden within the follicular compartment through this convergent route, independent of whatever specific upstream transcriptional lesion is responsible in a given case. Then, melatonin’s demonstrated capacity to reduce oxidative DNA damage and improve fertilization rate offers a plausible mechanism of clinical benefit that operates downstream of and largely independent from the specific circadian transcriptional defects detailed mechanistically in the earlier sections of this review, rather than requiring correction of those upstream defects directly [97].

This downstream positioning has a testable implication worth noting. If melatonin’s clinical benefit in obese IVF patients specifically derives from mitigating the convergent oxidative endpoint, rather than from correcting upstream BMAL1 transcriptional deficits, supplementation trials enrolling predominantly obese populations should show oxidative marker improvement of a magnitude at least comparable to that observed in non-obese populations, since the oxidative endpoint being targeted is proposed here as shared across the different upstream routes converging on it, obesity-driven and otherwise. Whether existing melatonin trials have been sufficiently powered or specifically designed to test this obesity-stratified prediction is addressed directly in the following subsection, which turns to the clinical trial evidence in more explicit detail.

### 7.3. Clinical Trial Evidence for Oocyte and Embryo Quality Outcomes

Beyond the mechanistic oxidative marker evidence discussed in the preceding subsection, several clinical trials have tested melatonin supplementation directly against oocyte and embryo quality endpoints in women undergoing IVF, and the pattern across these trials proves informative less for demonstrating uniform benefit than for revealing which specific outcome measures respond consistently and which do not, a distinction that matters considerably for how melatonin’s mechanism of action should be understood in light of the granulosa cell pathways detailed earlier in this review. A within-patient crossover design comparing an unsupplemented cycle against a subsequent melatonin-supplemented cycle in the same women found no significant difference in oocyte maturation rate, blastocyst rate, or the proportion of good-quality blastocysts between the two cycles, yet fertilization rate specifically improved substantially, rising from 35.1 percent in the unsupplemented cycle to 68.2 percent following melatonin supplementation among patients previously identified as having a low fertilization rate [10].

This selective improvement in fertilization rate without a corresponding improvement in maturation or blastulation rate is worth considering carefully rather than treated as an incidental detail of one trial’s results. Fertilization represents the specific developmental transition during which oocyte-intrinsic factors most directly determine outcome, since sperm penetration and pronuclear formation depend heavily on the oocyte’s cytoplasmic and mitochondrial competence at the moment of insemination, whereas maturation rate reflects an earlier developmental stage and blastocyst rate reflects a later one influenced substantially by post-fertilization embryonic genome activation and culture conditions independent of the oocyte’s original state [98]. A melatonin effect concentrated specifically at fertilization, without extending backward to maturation or forward to blastulation, is consistent with an antioxidant mechanism acting principally on the oocyte’s cytoplasmic and mitochondrial state at the point of fertilization itself, precisely the granulosa cell and oocyte mitochondrial pathways detailed extensively earlier in this review, rather than a broader effect on oocyte developmental potential considered across its full trajectory.

A separate randomized pilot trial specifically enrolling women with unexplained infertility, a population selected in part because these patients showed measurably blunted intrafollicular melatonin concentration and correspondingly elevated oxidative imbalance relative to fertile controls, tested two melatonin doses, 3 mg and 6 mg daily, against this same oxidative and fertilization framework [99]. Both doses ameliorated intrafollicular oxidative balance and improved oocyte quality relative to untreated patients, with this improvement translating into a modest increase in pregnancy and live-birth rate, though the trial’s authors themselves characterized this clinical translation as modest rather than dramatic, appropriately tempering the magnitude of benefit against the more robust improvement observed at the level of the oxidative and cellular markers measured directly. The absence of a clear dose-response advantage for the higher 6 mg dose over the 3 mg dose in this trial suggests a ceiling effect in melatonin’s antioxidant benefit within the follicular compartment, consistent with the follicle’s demonstrated capacity to actively concentrate melatonin independent of circulating dose described in the preceding subsection, since a tissue already accumulating hormone efficiently at a lower administered dose would be expected to show diminishing marginal benefit from further dose escalation.

An earlier randomized trial comparing melatonin-treated against untreated patients during IVF-embryo transfer found no difference in total oocyte number or total mature oocyte number between groups, yet the proportion of mature oocytes relative to total oocytes retrieved was significantly higher in the melatonin group, and the number of top-quality embryos was correspondingly higher despite fertilization rate itself showing no significant between-group difference in this particular trial [100]. This pattern, an improvement in oocyte maturation proportion and resulting embryo quality without a change in absolute oocyte yield or, in this case, fertilization rate specifically, differs somewhat from the fertilization-specific benefit observed in the Nishihara trial discussed above, and the discrepancy across trials is worth acknowledging directly rather than smoothing over, since it suggests melatonin’s clinical benefit may manifest at different points along the oocyte-to-embryo developmental sequence depending on the specific patient population studied, the baseline oxidative status of that population, and potentially the dosing protocol employed, rather than reflecting a single uniform mechanism producing an identical effect across every trial and every population.

### 7.4. Chronotherapeutic Hypotheses for Future Testing: Dose Timing, PCOS Populations, and Bariatric Surgery Scheduling

The clinical trials discussed in the preceding subsection administered melatonin as a straightforward daily oral dose without particular attention to circadian timing of administration relative to the patient’s own light-dark cycle, an omission worth noting explicitly given that melatonin’s physiological action depends inherently on timing rather than dose alone under normal endogenous secretion. Administering exogenous melatonin without regard to the patient’s habitual sleep-wake schedule risks introducing a degree of circadian noise into the very system the intervention is intended to support, a consideration seemingly absent from the trial designs reviewed above, each of which specified a total daily dose without reporting the time of administration relative to patients’ individual chronotype or habitual bedtime. Whether more carefully timed administration, synchronized to each patient’s endogenous melatonin onset rather than delivered as an arbitrary fixed-clock-time dose, would improve upon the modest benefits documented in the existing trials represents an untested refinement rather than an established finding, and one that follows logically from treating melatonin as a chronobiotic agent rather than simply as an antioxidant supplement administered without reference to time of day.

Route of administration is a related gap. Every trial discussed above used oral dosing, and none compared it against an alternative route. This matters mechanistically. Oral melatonin undergoes substantial first-pass hepatic metabolism, and its bioavailability is correspondingly low and variable. A cross-over pharmacokinetic study in healthy female volunteers found vaginal administration achieved close to complete bioavailability, well above the oral route [101]. Vaginal delivery would also reach the endometrium and the ovary through local vasculature, more directly than a swallowed dose. No trial has tested vaginal melatonin in women undergoing IVF, though, and the pharmacokinetic study cited here was conducted in healthy volunteers rather than a fertility population. Whether route of administration changes melatonin’s effect on oocyte or endometrial outcomes remains untested directly, and that gap should be stated plainly rather than left implicit.

Melatonin’s relevance to polycystic ovary syndrome specifically merits separate consideration given the BMAL1-SIRT1 mechanism detailed earlier in this review as a point of convergence between general obesity-related granulosa cell dysfunction and the more specific hormonal derangements characteristic of PCOS [98]. A trial enrolling PCOS patients specifically found melatonin supplementation during ovarian stimulation associated with a greater mean number of mature oocytes and a lower mean number of immature oocytes among patients with a prior history of poor oocyte quality, when melatonin was combined with inositol and folic acid relative to inositol and folic acid alone. This finding is consistent with, though does not directly confirm, the hypothesis that melatonin’s benefit in PCOS populations specifically might operate partly through the BMAL1-SIRT1 axis discussed earlier, given that inositol co-administration in this trial makes it difficult to attribute the observed benefit to melatonin in isolation, and the trial was not designed to measure BMAL1 or SIRT1 expression directly in a manner that would allow this specific mechanistic pathway to be confirmed or excluded [102].

Bariatric surgery raises a further chronotherapeutic question, though one that stays hypothetical rather than actionable at this stage. Whether weight-loss intervention timing relative to ovarian stimulation matters for patients considering IVF has not been tested in any trial. What follows is a research question, not a treatment recommendation. The adipocyte-level PPAR-γ/SLC1A5/BMAL1 mechanism detailed early in this review carries a specific implication for this sequencing question, as reversing the epigenetic silencing of Bmal1 in obese adipose tissue requires restoring the glutamine and methionine flux through SLC1A5. Since SLC1A5 expression itself depends on PPAR-γ activity recovering from both the metabolic and inflammatory suppression detailed earlier in this review, the resolution of adipocyte clock disruption following bariatric surgery would be expected to lag behind the resolution of the body mass index itself, given that PPAR-γ activity, inflammatory cytokine burden, and epigenetic marks depend on time courses that do not necessarily track weight loss in a simple, linear fashion [41]. None of this should guide current clinical decisions. Dose timing, PCOS-specific protocols, and surgery-to-stimulation intervals are directions for future trials to test, not adjustments to make in practice today.

## 8. Discussion

The evidence gathered in this review is consistent with a candidate integrative model in which obesity-associated disruption of the adipocyte clock may contribute to downstream changes in granulosa cells and the endometrium through shared hormonal and inflammatory signals, potentially converging on oxidative stress in the follicle, impaired steroid production, and a shifted window of implantation in the uterus. This sequence is proposed as a testable framework rather than an established causal chain; no single study has followed it from start to finish in one experiment, and it has been pieced together here from separate literature that rarely speaks to each other directly. The clinical picture, especially in donor-oocyte studies, does not agree from one paper to the next. One plausible, testable explanation for that disagreement is a sample-size and severity problem rather than a genuine absence of biological effect, although this explanation has not been directly tested and alternative accounts cannot be excluded. Melatonin trials give some support for a downstream fix, but none of them repair the core transcriptional defect at its source. Not every clock gene behaves the same way under this model. Section 3.1 traces which genes attenuate together with BMAL1 in obesity and which do not. Section 5.2 shows that PER2 performs the role opposite that of BMAL1, and that its selective silencing is required for healthy decidualization and is not a sign of damage. The mechanism proposed here is BMAL1-centered because the evidence points that way, not because CLOCK and PER were left out.

Wang et al. (2022) showed where the chain starts. In obese white adipose tissue, PPAR-γ activity falls, and this blocks SLC1A5-driven uptake of glutamine and methionine into the adipocyte [16]. Without these two amino acids, the cell cannot generate the acetyl-CoA and S-adenosylmethionine needed to keep the Bmal1 promoter open for transcription. The clock in fat tissue becomes quiet as a direct result. This finding matters for the rest of the review because it gives a starting point with a clear molecular cause, not just a correlation between obesity and a flattened rhythm. Chu et al. (2019) picked up the thread from here [32]. Their work showed that BMAL1 regulates Lepr expression directly in granulosa cells, working through leptin receptor signaling to control estrogen output. Since leptin rises in direct proportion to the fat mass that Wang et al. describe as circadian-disrupted, Chu et al.’s finding gives leptin a specific molecular door into the ovary rather than leaving it as a vague marker of obesity with no defined mechanism [32]. Read together, these two papers turn “obesity affects fertility” into something far more concrete: a broken adipocyte clock raises leptin, and leptin then meets a granulosa cell whose own sensitivity to leptin depends on a clock gene that the very same obesity has already weakened.

Kawamura et al. (2024) moved the story one step further into the granulosa cell itself [7]. Silencing BMAL1 in human luteinized granulosa cells lowered CYP11A1, CYP19A1, STAR, and ESR2 expression all at once, hitting cholesterol transport, the rate-limiting steroid synthesis step, aromatase, and the estrogen receptor together rather than any single gene alone. Read next to Chu et al. (2019), this suggests two separate routes converge on the same granulosa cell steroidogenic failure: one running through Lepr-mediated leptin signaling, and one running through BMAL1’s own direct grip on the steroidogenic enzymes themselves [32]. A granulosa cell exposed to both a disrupted leptin signal and a weakened BMAL1 transcriptional program at the same time would be expected to show a more severe steroidogenic deficit than either mechanism working alone, which may explain why granulosa cell dysfunction in obesity has proven so consistent and so hard to reverse through any single intervention.

Lv et al. (2019) and Muter et al. (2015) together tell a more complicated story about the endometrium, one that resists a simple “BMAL1 loss equals damage” reading [8,62]. Lv et al. found that silencing BMAL1 in decidualizing stromal cells lowered TIMP3 expression and caused trophoblast cells to overinvade the decidua, and they linked reduced BMAL1 in decidual tissue directly to recurrent miscarriage in patients [8]. Muter et al., working in a different but related system, found that normal decidualization itself switches off clock oscillation on purpose, through a specific and selective loss of CLOCK binding at the PER2 promoter, while binding at other clock promoters stays intact [62]. Put next to each other, these two studies say something worth pausing on: losing rhythm is not automatically bad for the endometrium. Some loss of rhythm is required and healthy, timed precisely to decidualization. What Lv et al. describe as pathological is not rhythm loss in general; instead, it is BMAL1 failing at the wrong moment, or losing its restraint on TIMP3 specifically, rather than losing its oscillation as such [8]. No study so far has compared decidualizing cells from obese and lean women at matched stages of differentiation to see whether obesity disrupts this careful, controlled process or instead imposes a different, untimed kind of disruption on top of it.

Zhou et al. (2025) added a further route running in parallel to the one described by Lv et al. (2019) [8,66]. Their work traced a BMAL1/REV-ERB feedback loop controlling BMP2/4 and GDF10/15, growth factors involved in decidual transformation and trophoblast signaling, working through a mechanism distinct from the TIMP3-invasion pathway. The same pattern of more than one converging route shows up again here, mirroring what Kawamura et al. (2024) and Chu et al. (2019) already showed on the ovarian side of this review [7,32]. Sen and Sellix (2016) and Summa et al. (2012) sit underneath both of these tissue-specific stories as broader support [103,104]. Sen and Sellix reviewed how environmental circadian disruption, independent of the obesity-specific mechanisms detailed above, damages reproductive function at the level of the hypothalamic-pituitary-gonadal axis, while Summa et al. showed directly in mice that shifting the light–dark cycle experimentally reduces pregnancy success [103,104]. Neither paper mentions obesity or BMAL1 in adipose tissue specifically, but both establish that circadian disruption alone, through any cause, is sufficient to damage reproduction, which lends independent weight to the more detailed obesity-specific mechanism built from Wang et al. (2022) onward [16].

The clinical literature testing whether all of this actually shows up as endometrial dysfunction in obese patients is the messiest part of this review, and it deserves to be read carefully rather than summarized as a simple yes-or-no. Bellver et al. (2013), working with 9587 donor-oocyte cycles, found implantation and live-birth rates falling step by step across four BMI categories, while fertilization and cleavage rates in the lab stayed flat across the same groups [87]. This detail matters; since laboratory-stage embryo quality did not differ by BMI, the decline in live birth points toward the endometrium rather than the oocyte, exactly where Lv et al. (2019) and Zhou et al. (2025) would place the effect [8,66]. Bellver et al. (2021) then confirmed this directly, using receptivity array testing to show a displaced window of implantation in obese women, with the displacement growing worse specifically at the more severe obesity classes [105]. Set against this, Styne-Gross et al. (2005), Wattanakumtornkul et al. (2003), Setton et al. (2020), and Moreta et al. (2022) each found no BMI effect on implantation, using progressively tighter designs: sibling-oocyte pairing in Setton et al., and ploidy-restricted analysis in Moreta et al. [4,84,85,86]. The obesity-class pattern that Bellver et al. (2021) documented offers one possible way to reconcile this split, among explanations that remain to be tested directly [105]. If the mechanisms from Wang et al. (2022) and Chu et al. (2019) need a certain severity of adiposity before producing a measurable clinical effect, cohorts weighted toward mild overweight and class I obesity, which several of the null-finding studies appear to be, would be poorly positioned to detect an effect concentrated specifically in class II and III obesity [16,32]. None of the null-finding studies broke their obese group down by severity in a way that could test this directly, leaving the question open rather than resolved by the existing data.

Tamura et al. (2008), Nishihara et al. (2014), Batıoğlu et al. (2012), and Espino et al. (2019) show melatonin’s clinical footprint landing in a different place almost every time, rather than improving every stage of oocyte and embryo development uniformly [9,10,99,100,104]. Tamura et al. established the foundation this entire line of evidence rests on: intrafollicular melatonin runs inversely to 8-hydroxy-2′-deoxyguanosine, the oxidative DNA damage marker, and raising melatonin through oral supplementation lowered this marker while improving fertilization rate [9]. Nishihara et al. then found a large jump in fertilization rate specifically, from 35.1 to 68.2 percent, with no change in maturation rate or blastocyst rate in the same patients [10]. Batıoğlu et al. found close to the opposite pattern: better oocyte maturation proportion and more top-quality embryos, but no differences in fertilization rate between groups [100]. Espino et al., studying women chosen specifically for low baseline follicular melatonin, found improved oxidative balance translating into only a modest gain in pregnancy rate. Four trials, four somewhat different outcome patterns [99]. That variation fits more comfortably with melatonin acting on a shared oxidative endpoint that different patient populations reach through different upstream routes, consistent with the multiple convergent granulosa cell pathways described earlier in this review, than with one single mechanism producing an identical effect regardless of population. None of these four trials, however, reported results split by BMI, so whether melatonin’s benefit is larger, smaller, or unchanged in obese patients specifically remains untested by the existing trial evidence, despite obesity sitting at the center of the mechanistic argument this review has built.

This review stays narrative rather than systematic. No formal bias scoring was applied, and no protocol was registered ahead of time, a choice that follows from how wide the topic runs, spanning adipocyte biology, ovarian physiology, and endometrial receptivity together, rather than from a gap in the design itself. The mechanistic chain built here links adipocyte, granulosa cell, and endometrial evidence drawn from separate experimental systems and, in some cases, separate species entirely. No single study confirms the whole route from Wang et al.’s (2022) adipocyte-level PPAR-γ/SLC1A5/Bmal1 mechanism through to Bellver et al.’s (2021) clinical receptivity findings within one cohort or one experimental design. The clinical studies discussed in Section 6 are mostly retrospective, use different BMI cutoffs to define comparison groups, and were run across a span of nearly two decades during which IVF laboratory practice itself changed considerably [16,105].

Several well-established, non-circadian mechanisms account for much of the reduced fertility observed in obese women undergoing IVF, and they are presented here as major explanatory frameworks in their own right, not as a subsidiary counterpoint to the BMAL1-centered model developed throughout this review. Petersen and Shulman (2018) make a strong case that insulin resistance alone explains much of the metabolic disturbance seen in obese women undergoing IVF, through pathways that never touch a clock gene [30]. Zatterale et al. (2020) trace that insulin resistance back to chronic low-grade adipose inflammation, again without any need for BMAL1 as an intermediate [28]. Wu et al. (2010) showed that a high-fat diet triggers lipotoxicity in cumulus-oocyte complexes directly, through lipid accumulation, ER stress, and reduced mitochondrial membrane potential, independent of any circadian mechanism [106]. Yan et al. (2025) and Li et al. (2025) document mitochondrial dysfunction in granulosa cells extensively in PCOS and obesity, and neither paper mentions circadian genes at all [46,55]. Varra et al. (2025) frame oxidative stress, treated here as a downstream convergence point for BMAL1 loss, as a more direct consequence of excess adiposity itself [96]. None of this makes the BMAL1 model wrong. However, this means that BMAL1 dysregulation should be viewed as a candidate integrative mechanism operating alongside insulin resistance, inflammation, lipotoxicity, mitochondrial dysfunction, and oxidative stress, rather than as an established upstream cause standing above them. Where this review adds value is in tying these separate threads to a shared upstream clock defect and a testable timing hypothesis. This review does not replace insulin resistance, inflammation, lipotoxicity, or oxidative stress as explanations; in places, it may simply reflect or converge with them rather than drive them.

The gaps identified across this review point toward specific next steps rather than a general call for more research. Direct measurement of BMAL1, REV-ERB, and downstream growth factor expression in granulosa cells and endometrium, broken down by obesity class, following the receptivity array approach used by Bellver et al. (2021), would test whether the severity threshold proposed here to reconcile the donor-oocyte literature reflects a genuine circadian mechanism rather than some other obesity-linked factor running in parallel [105]. Comparing decidualizing stromal cells from obese and lean women at matched differentiation stages would test whether obesity disrupts the carefully timed clock silencing that Muter et al. (2015) described, and whether it achieves such through the same TIMP3 route that Lv et al. (2019) documented or through some separate mechanism entirely [8,62].

A paired design could test this directly. Endometrial stromal cells would be isolated from obese and normal-weight women, matched by BMI category on one side and by menstrual cycle stage on the other, so that neither variable confounds the comparison. Both sets of cells would be decidualized synchronously in vitro under an identical induction protocol. PER2 and BMAL1 expression would then be tracked together over the course of decidualization, not at a single endpoint, since the physiological pattern described by Muter et al. (2015) is a timed silencing event, not a fixed expression level [62]. The comparison that matters is not whether obese cells show lower PER2 or BMAL1 at one time point, but whether the timing and completeness of PER2 silencing is delayed, incomplete, or shifted relative to the lean-cell pattern, and whether BMAL1 loss in the obese cells tracks the same TIMP3 route Lv et al. (2019) documented or departs from it [8]. This would distinguish obesity disrupting the physiological silencing sequence itself from obesity simply adding a separate, unrelated BMAL1 lesion on top of normal decidualization timing. A melatonin trial built specifically to compare obese against non-obese participants, ideally using circadian-timed rather than fixed-clock dosing, would close the gap left open by Nishihara et al. (2014), Tamura et al. (2008), Batıoğlu et al. (2012), and Espino et al. (2019) together, and would offer the clearest available test of whether the model proposed throughout this review leads anywhere useful in the clinic rather than remaining a plausible but untested synthesis [9,10,99,100,104].

## 9. Conclusions

Obesity does not damage fertility through a single defect in the oocyte or a single defect in the endometrium. This review has proposed a more specific candidate model: obesity-associated disruption of the adipocyte clock may propagate through leptin and inflammatory signaling to affect the ovary and the uterus, each carrying local BMAL1-dependent machinery that could be vulnerable to this disruption in slightly different ways. This model is offered as an integrative hypothesis that organizes the mechanistic literature reviewed here, not as an established causal sequence. In this proposed model, the sequence would start with PPAR-γ suppression cutting off the glutamine and methionine supply that keeps Bmal1 transcription active in fat tissue. From there, it would reach the ovary through two separate routes, leptin receptor signaling and direct loss of steroidogenic enzyme transcription, both converging on the same granulosa cell failure. A parallel but more delicate story is proposed for the endometrium, where losing rhythm is not simply harmful in itself but may become harmful when it happens at the wrong time or removes the wrong restraint, potentially disrupting trophoblast invasion control and growth factor signaling together rather than through one single lesion.

The clinical evidence testing this model directly remains genuinely split. This review has proposed a sample-size and severity explanation for that split as one plausible, testable account; it is not presented as the preferred or established interpretation, and it should be weighed against competing explanations, including protocol heterogeneity and confounding across the retrospective cohorts discussed in Section 6. Consistent with this hypothesis, the largest available donor-oocyte cohort found a dose-dependent decline in live birth across BMI categories, later corroborated by direct measurement of a displaced window of implantation concentrated specifically in more severe obesity. Several smaller and more tightly controlled studies found no such effect, in cohorts weighted toward milder obesity and, in some cases, too small to detect an effect of the size seen in the larger series. Melatonin offers a genuine but partial answer, working through the oxidative pathway this review has traced back to disrupted mitochondrial biogenesis and mitophagy in the granulosa cell, without correcting the BMAL1 transcriptional defect at its source.

Taken as a whole, the mechanisms described throughout this review turn a long-standing and largely unresolved clinical debate, whether obesity harms the oocyte or the endometrium, into a question that may not need to be answered as an either-or. Both compartments appear to sit downstream of the same upstream lesion, reached through different molecular routes but originating from the same broken adipocyte clock. What remains untested is whether this account is correct in the way this review proposes rather than merely consistent with it; no study has yet measured BMAL1 expression across adipose tissue, granulosa cells, and endometrium in the same obese patients, and no melatonin trial has yet been powered specifically to detect an obesity-dependent difference in response. Until these direct tests are carried out, the model offered here stands as a plausible synthesis rather than a demonstrated mechanism. Closing this gap is the clearest next step for anyone working at the intersection of circadian biology and reproductive medicine.

Three limitations run through this review and deserve to be named together, not scattered across earlier sections. First, much of the causal mechanistic detail rests on mice and immortalized cell lines, not human tissue in vivo. Knockdown and overexpression experiments in KGN and HGL5 cells, and Bmal1 knockout mice, establish causality within those systems. They do not establish that the same causal chain runs the same way in a living human ovary or endometrium. Second, the clinical literature carries confounding elements that this review has flagged but not resolved. Donor-oocyte studies use different stimulation and endometrial preparation protocols, different BMI cutoffs, and span two decades of changing laboratory practice, and few control for the recipient hormone-dosing confound discussed in Section 6.1. Third, no studies have measured BMAL1 expression longitudinally in human reproductive tissue. Every human data point in this review is a single time point snapshot, not a rhythm tracked over hours or across a stimulation cycle. Without that longitudinal measurement, amplitude loss and phase shift cannot be told apart in the tissues that matter most for this review’s argument. These three gaps do not undermine the case made here; instead, they mark the difference between a plausible model and a demonstrated one.

## Figures and Tables

**Table 1:** Summary of molecular mechanisms by which obesity-driven circadian disruption propagates from adipose tissue through ovarian granulosa cells to the endometrium, drawn from Section 3, Section 4 and Section 5. Mechanisms are organized by tissue compartment to illustrate convergence of multiple independent pathways on shared functional endpoints (steroidogenic failure, oxidative stress, and displaced endometrial receptivity).

Tissue	Molecular Mechanism	Functional Consequence
Adipose tissue [16]	Reduced PPAR-γ activity suppresses SLC1A5-mediated glutamine/methionine uptake, lowering H3K27ac and H3K4me3 marks at the Bmal1 promoter	Flattened BMAL1 amplitude in obese white adipose tissue
Granulosa cells (porcine model) [32]	BMAL1 regulates Lepr, Cyp19a1, Cyp11a1, and Fshr expression; BMAL1 knockdown blocks leptin-driven upregulation of these genes	Impaired leptin-dependent estradiol synthesis
Granulosa cells (human) [7]	BMAL1 loss reduces CYP11A1, CYP19A1, STAR, and ESR2 transcription	Impaired steroidogenesis and reduced estradiol output
Endometrial stroma [8]	BMAL1 silencing reduces TIMP3 expression during decidualization	Trophoblast overinvasion; associated with recurrent miscarriage
Endometrial stroma [62]	Selective loss of CLOCK binding at the PER2 promoter during decidualization	Physiological silencing of clock oscillation coordinating decidual transformation
Endometrium [66]	BMAL1/REV-ERB loop regulates BMP2/4 and GDF10/15 transcription	Altered growth-factor signaling relevant to implantation
Endometrium (clinical) [76]	Obesity associated with a displaced window of implantation on receptivity array testing	Endometrial receptivity defects independent of oocyte quality

**Table 2:** Competing explanatory frameworks for obesity-related IVF failure and their predicted signature in the donor-oocyte model. The oocyte-centered and endometrium-centered hypotheses represent the two dominant interpretations in the existing literature, while the circadian synthesis proposed in this review offers a unifying account that additionally predicts a severity threshold, potentially explaining the divergent findings examined in Section 6.2 and Section 6.3.

Framework	Proposed Primary Site of Defect	Underlying Mechanism	Predicted Donor-Oocyte Model Result	Predicted Sensitivity to Obesity Severity
Oocyte-centered hypothesis	Oocyte/granulosa cell	Lipotoxicity, mitochondrial compromise, oxidative damage originating in the follicle	No recipient BMI effect (defect resides in donor’s own oocyte, not the recipient’s uterus)	Would track donor BMI, not recipient BMI
Endometrium-centered hypothesis	Endometrial stroma/receptivity	Altered steroid signaling, chronic low-grade inflammation in the uterine environment	Recipient BMI effect present, independent of donor oocyte quality	Would track recipient BMI directly
Circadian synthesis (this review)	Both compartments, via a shared upstream lesion	Adipocyte BMAL1 disruption propagating through leptin/inflammatory signaling to both granulosa and endometrial clocks	Recipient BMI effect present, but only above a severity threshold	Effect concentrated in class II/III obesity rather than uniform across all overweight/obese categories

## Data Availability

No new data were created or analyzed in this study. Data sharing is not applicable to this article.

## Abbreviations

**Abbreviation**	**Full term**
BMAL1	Brain and Muscle ARNT-Like 1
CLOCK	Circadian Locomotor Output Cycles Kaput
PER	Period circadian regulator
CRY	Cryptochrome
REV-ERBα	Nuclear receptor subfamily 1, group D, member 1 (NR1D1)
PPAR-γ	Peroxisome proliferator-activated receptor gamma
SLC1A5	Solute carrier family 1 member 5
SAM	S-adenosylmethionine
TNF-α	Tumor necrosis factor alpha
Lepr	Leptin receptor
CYP11A1	Cholesterol side-chain cleavage enzyme
CYP19A1	Aromatase
STAR	Steroidogenic acute regulatory protein
ESR2	Estrogen receptor beta
SIRT1	Sirtuin 1
UPRmt	Mitochondrial unfolded protein response
ROS	Reactive oxygen species
8-OHdG	8-hydroxy-2′-deoxyguanosine
HIF-1α	Hypoxia-inducible factor 1-alpha
BNIP3	BCL2/adenovirus E1B 19 kDa protein-interacting protein 3
TIMP3	Tissue inhibitor of metalloproteinases 3
BMP2/4	Bone morphogenetic protein 2/4
GDF10/15	Growth differentiation factor 10/15
HOXA10	Homeobox A10
IVF	In vitro fertilization
BMI	Body mass index
PCOS	Polycystic ovary syndrome
